# The distinct ripening processes in the reproductive and non-reproductive parts of the fig syconium are driven by ABA

**DOI:** 10.1093/jxb/ery333

**Published:** 2018-08-31

**Authors:** Kumar Lama, Sharawan Yadav, Yogev Rosianski, Felix Shaya, Amnon Lichter, Lijuan Chai, Yardena Dahan, Zohar Freiman, Reut Peer, Moshe A Flaishman

**Affiliations:** 1 Institute of Plant Sciences, Agricultural Research Organization, Bet-Dagan, Israel; 2 The Robert H. Smith Institute of Plant Sciences and Genetics in Agriculture, Faculty of Agriculture, Food and Environment, The Hebrew University of Jerusalem, Rehovot, Israel; 3 Institute of Postharvest and Food Sciences, Agricultural Research Organization, Bet-Dagan, Israel

**Keywords:** Abscisic acid (ABA), ethylene, *FcACS4*, *FcNCED2*, *Ficus carica*, fruit ripening, on-tree treatment

## Abstract

The common fig bears a unique closed inflorescence structure, the syconium, composed of small individual drupelets that develop from the ovaries, which are enclosed in a succulent receptacle of vegetative origin. The fig ripening process is traditionally classified as climacteric; however, recent studies have suggested that distinct mechanisms exist in its reproductive and non-reproductive parts. We analysed ABA and ethylene production, and expression of ABA-metabolism, ethylene-biosynthesis, MADS-box, NAC, and ethylene response-factor genes in inflorescences and receptacles of on-tree fruit treated with ABA, ethephon, fluridone, and nordihydroguaiaretic acid (NDGA). Exogenous ABA and ethephon accelerated fruit ripening and softening, whereas fluridone and NDGA had the opposite effect, delaying endogenous ABA and ethylene production compared to controls. Expression of the ABA-biosynthesis genes *FcNCED2* and *FcABA2*, ethylene-biosynthesis genes *FcACS4*, *FcACOL*, and *FcACO2*, *FcMADS8*, *14*, *15*, *FcNAC1*, *2*, *5*, and *FcERF9006* was up-regulated by exogenous ABA and ethephon. NDGA down-regulated *FcNCED2* and *FcABA2*, whereas fluridone down-regulated *FcABA2*; both down-regulated the ethylene-related genes. These results demonstrate the key role of ABA in regulation of ripening by promoting ethylene production, as in the climacteric model plant tomato, especially in the inflorescence. However, increasing accumulation of endogenous ABA until full ripeness and significantly low expression of ethylene-biosynthesis genes in the receptacle suggests non-climacteric, ABA-dependent ripening in the vegetative-originated succulent receptacle part of the fruit.

## Teaser

ABA produced in the fig fruit triggers ethylene production in the inflorescence and induces non-climacteric ripening in the receptacle tissue.

## Introduction

Fruit ripening involves the well-orchestrated coordination of several regulatory steps in both climacteric and non-climacteric fruit, together with strong metabolic and physiological changes (Kumar *et al.*, 2014). Fig fruit (*Ficus carica*) are categorized as climacteric; they show a rise in respiration rate and ethylene production at the onset of their ripening phase (Marei and Crane, 1971; Chessa *et al.*, 1992), similar to that of the climacteric tomato, apple, and mango fruit (Giovannoni, 2001; Adams-Phillips *et al.*, 2004; Zaharah *et al.*, 2012). However, unlike climacteric fruit, figs harvested before they are fully ripe never complete their ripening process to reach commercially desirable parameters of size, color, flavor, and texture (Flaishman *et al.*, 2008). This challenges the idea that fig fruit are climacteric. In addition,1-methylcyclopropene (1-MCP), which blocks the effects of ethylene during ripening, increases ripening-related ethylene production in fig fruit following pre- or post-harvest application in an unexpected auto-inhibitory manner (Sozzi *et al.*, 2005; Owino *et al.*, 2006; Freiman *et al.*, 2012). A molecular study of ethylene-related genes in figs (Freiman *et al.*, 2015) showed that *FcERF12185*, an ethylene signal-transduction gene, might be responsible for the non-climacteric auto-inhibition of ethylene production in the fruit. Unlike most fig ethylene-response factor (ERF) genes, *FcERF12185* expression does not increase during ripening, and is induced upon 1-MCP treatment (Freiman *et al.*, 2015).

It has been suggested that the plant hormone abscisic acid (ABA) regulates fruit ripening and senescence in both climacteric and non-climacteric fruit (Zhang *et al.*, 2009*a*, 2009*b*; Sun *et al.*, 2012; Leng *et al.*, 2014). In climacteric fruit, endogenous ABA levels increase before the onset of ripening and subsequently decrease until the fruit is fully ripe. However, in non-climacteric fruit, ABA levels increase from maturation to harvest (Setha, 2012; Leng *et al.*, 2014).

The fig fruit bears a unique closed inflorescence structure, the syconium. This closed inflorescence produces an aggregate fruit, which is composed of small individual drupelets that develop from the ovaries enclosed in the receptacle (Storey *et al.*, 1977). Development of female fruit in the common fig consists of three phases: phase I is characterized by rapid growth in fruit size; in phase II, the fruit remains nearly the same size, color, and firmness; in phase III, ripening occurs, with fruit growth, color change, softening, and alteration of the pulp texture to an edible state (Flaishman *et al.*, 2008). Endogenous ABA is reported to increase as the ripening phase progresses (Rosianski *et al.*, 2016*a*). The endogenous ABA content in the fruit is determined by the dynamic balance between its biosynthesis and catabolism. Zeaxanthin epoxidase (ZEP), 9-cis-epoxycarotenoid dioxygenase (NCED), short-chain alcohol dehydrogenase (ABA2), and abscisic aldehyde oxidase participate in ABA biosynthesis, while ABA-8’-hydroxylase (ABA8OX) and ABA-glucosyltransferase participate in its catabolism (Schwartz *et al.*, 2003; Setha *et al.*, 2005; Taylor *et al.*, 2005; Jiang and Hartung, 2008; Seo and Koshiba, 2011; Leng *et al.*, 2014; Rosianski *et al.*, 2016*a*).

The effect of ABA on ethylene biosynthesis, fruit ripening, and senescence has been extensively studied in the model plant tomato. Exogenous ABA treatment increases ABA content in tomato fruit, and induces the expression of ethylene-biosynthesis genes, namely 1-aminocyclopropane-1-carboxylic acid (ACC) synthase (*ACS*) and ACC oxidase (*ACO*). On the other hand, treatment of tomato fruit with the ABA inhibitors fluridone or nordihydroguaiaretic acid (NDGA) inhibits *ACS* and *ACO* and delays ripening and softening (Zhang *et al.*, 2009*b*; Mou *et al.*, 2016). In particular, a significant reduction in the activity of *SlNCED1*, a key gene in tomato ABA biosynthesis, by RNAi, reduces the expression of genes encoding major cell wall-catabolic enzymes, and increases the accumulation of pectin during ripening, which leads to a significant extension of the shelf-life of climacteric tomato fruit (Sun *et al.*, 2012). Similarly, exogenous ABA application significantly promotes the ripening process of the non-climacteric strawberry fruit, whereas fluridone inhibits it. Down-regulation of the ABA-biosynthesis gene *FaNCED1* in strawberry significantly decreases ABA levels and produces non-colored/unripe fruit (Jia *et al.*, 2011). Moreover, studies in apple, banana, grape, and sweet cherry have shown that exogenous application of ABA enhances fruit ripening by up-regulating ethylene production, as well as anthocyanin and sugar accumulation (Jiang *et al.*, 2000; Lara and Vendrell, 2000; Ban *et al.*, 2003; Jiang and Joyce, 2003; Giribaldi *et al.*, 2010; Luo *et al.*, 2014). In fig fruit, genes of the ABA biosynthesis and catabolism pathways have been isolated and their expression characterized during ripening (Rosianski *et al.*, 2016*a*).In addition, Freiman *et al.* (2015) performed a comprehensive study focusing on ethylene-biosynthesis, MADS-box, and ethylene signal-transduction genes during natural ripening and their interactions with 1-MCP. However, the relationship between ABA and ethylene during the onset of fruit ripening in fig remains to be fully elucidated.

The plant-specific NAC family of proteins containing the NAC domain is one of the largest transcription factor families in plants. An increasing number of NAC genes are being identified and studied in both monocotyledonous and dicotyledonous plants . In tomato, 74 NAC or NAC-like genes belonging to 12 subfamilies have been identified, with high expression of *SlNAC4–9* being found during fruit development and ripening (Kou *et al.*, 2014). *SlNAC1* has a broad influence on tomato fruit ripening, and its over-expression during ripening has been found to be regulated through both ethylene-dependent and ABA-dependent pathways (Ma *et al.*, 2014). Reduced expression of *SlNAC4* by RNAi in tomato results in delayed fruit ripening, with suppressed chlorophyll breakdown and decreased ethylene synthesis being mediated mainly through reduced expression of system-2 ethylene-biosynthesis genes, and with carotenoids being reduced via alteration of the flux of the carotenoid pathway (Zhu *et al.*, 2014). In addition, *SlNAC4* is down-regulated by ABA treatment while *SlNAC5*, *6*, *7*, and *9* are up-regulated (Kou *et al.*, 2014). Involvement of NAC genes in banana fruit ripening was found via interactions with ethylene-signaling components (Shan *et al.*, 2012). In figs, 27 NAC genes have been identified during fruit ripening (Freiman *et al.*, 2014); however, their roles during this process are still unknown.

In the present work, we used exogenous ABA, ethephon, and the ABA inhibitors fluridone and NDGA to characterize the effects of ABA on fig fruit ripening. The expression levels of ABA- and ethylene-biosynthesis genes were determined, together with those of ripening-associated transcription factors and signal-transduction genes. The levels of endogenous ABA and ethylene were also quantified to determine how ABA triggers ethylene production to start the ripening process. This study also examined the genes that likely to be induced during on-tree fruit ripening. The non-climacteric ripening behavior of fig fruit associated with ABA is also discussed.

## Materials and methods

### Plant material and sample preparation

Fruit of common fig (*Ficus carica* L.) cv. Brown Turkey, 37 mm in diameter with a greenish-yellow ostiole color (just before the rapid increase in endogenous ABA production and the start of ethylene production), were selected for on-tree treatment with ABA, ethephon, fluridone, and NDGA in a fig orchard located at the Agricultural Research Organization – Volcani Center, Israel. Treatments were applied by injecting 1 ml of the following into the fruit through the ostiole with a plastic syringe: 1.89 mM ABA (Valent Bioscience Corporation, USA), 0.7 mM ethephon (Ishihara Sangyo Kaisha, Ltd., Japan), 0.15 mM NDGA, or 0.2 mM fluridone (both Sigma-Aldrich). ABA and ethephon were injected once at time 0, whilst NDGA and fluridone were injected three times at 12-h intervals starting at time 0. The size and ostiole color of the fruit used in the experiments and the treatment concentrations were selected on the basis of several preliminary trials (data not shown). Fruit treated with ddH_2_O served as the control group and ethephon-treated fruit served as a positive control.

Three separate experiments were performed: (i) ABA and ethephon treatment; (ii) fluridone treatment; and (iii) NDGA treatment. These trials were conducted during the summers of 2015 and 2016 (July–August), with average day and night temperatures of 32 °C and 24 °C, respectively. For fruit treated with ABA and ethephon, samples were collected at 0, 12, 24, 48, 72, and 96 h after treatment (HAT). Samples for the NDGA and fluridone treatments were collected at 0, 24, 32, 48, 72, and 96 HAT. In total, 600 fruit were treated in each experiment and physiological changes were examined. Nine fruit were harvested at each time interval for molecular characterization, divided into three biological replicates consisting of three fruit each. Fruit receptacle and inflorescence tissues were collected separately and stored at –80ºC until further analysis.

### Fruit diameter and firmness

Fruit fresh weight was determined immediately on sampling. Width was measured using a standard caliber, and was recorded as the maximal horizontal diameter. Fruit firmness was measured using an Inspekt Table Blue universal testing machine with 5 kN capacity (Hegewald & Peschke MTP, Nossen, Germany), and expressed in Newtons as F(N), the energy used to deform the fruit to 5% of its diameter, as in Rosianski *et al.* (2016*b*).

### Measurement of ethylene production

Ethylene production was determined according to Freiman *et al.* (2012) in a total of seven fruit at each sampling time. Individual fruit were enclosed in a 0.75-l airtight glass jar for 2 h at room temperature (20 °C)and ethylene was quantified using a Varian 3300 GC instrument with a flame-ionization detector (FID) (Varian Inc., CA, USA), a stainless-steel column (length 1.5 m, outside diameter 3.17 mm, internal diameter 2.16 mm) packed with HayeSep T, particle size 0.125–0.149 mm (Alltech Associates Inc., IL, USA), and helium as the carrier gas (5 ml min^–1^).

### ABA extraction and quantification

Frozen receptacles or inflorescences were ground into powder and 0.5-g samples were dissolved in 3 ml of 80% acidified methanol and 1% acetic acid in 15-ml tubes. d4-ABA (7 µl of a 10× dilution) was added as an internal standard and, after vortexing, the mixture was stored at 4 °C for 2 h. Samples were centrifuged at 1510 *g* for 30 min at 4 °C and the upper phase was transferred to a new tube. The pellet was extracted again by adding 3 ml of 80% acidified methanol and storing overnight at 4 °C, before further centrifuging. The separated upper phases were combined and then evaporated at 40 °C down to 200 µl and stored at –20 °C for further analysis.

 LC–MS analyses were conducted using a UPLC-Triple Quadrupole-MS (Waters Xevo TQ, MS, USA). Samples were centrifuged for 15 min at 13 362 *g* and 1 µl of supernatant was injected into the LC–MS instrument. Separation was performed on a 2.1 × 100 mm^2^, 1.7-µm ACQUITY UPLC BEH C18 column with a VanGuard pre-column (BEH C18 1.7 µm, 2.1 × 5 mm^2^). The chromatographic and MS parameters were as follows: the mobile phase consisted of water (phase A) and acetonitrile (phase B), both containing 0.1% formic acid, in gradient-elution mode. The solvent gradient program was as follows: 5% to 95% A over 0.1 min, 25% to 75% A over 2 min, 35% to 65% A over 2.5 min, 40% to 60% A over 3 min, held at 95% to 5% A for 4 min. At the end of the gradient, the column was washed with 95% B (3 min) and re-equilibrated to initial conditions for 3 min. The flow rate was 0.3 ml min^–1^, and the column temperature was kept at 35 °C. All of the analyses were performed using the ESI source in positive ion mode with the following settings: capillary voltage 3.5 kV, cone voltage 26 V, desolvation temperature 350 °C, desolvation gas flow 650 l h^–1^, source temperature 150 °C. Quantitation was performed using multiple reaction monitoring (MRM) acquisition by monitoring 247/187, 247/173 (RT=3.93, dwell time of 78 ms for each transition) for ABA and 251/191, 251/177 (RT=3.93, dwell time of 78 ms) for d4-ABA (used as an internal standard). The LC–MS data were acquired using the MassLynx V4.1 software (Waters).

### RNA extraction and cDNA synthesis

Total RNA was extracted according to Jaakola *et al.* (2001). The RNA concentration was determined in a Nanodrop ND-1000 spectrophotometer, and its integrity was checked by running 1 μl in a 1% (w/v) agarose gel stained with Bromophenol Blue. Total RNA was digested with RQ-DNase (Promega). Complementary DNA was synthesized, using Oligo-dT primers, using a VERSO cDNA kit (Thermo Scientific).

### High-throughput real-time quantitative PCR

High-throughput real-time qPCR was performed on a BioMark 96.96 Dynamic Array with TaqMan Gene Expression Assays (Applied Biosystems) at the Weizmann Institute of Science (Israel). Three biological replicates were used for each treatment. Primers were designed with the Primer3 software, and synthesized by Metabion (Germany) and Hylabs (Israel) (see Supplementary Table S1 at *JXB* online). The expression levels of the target genes were normalized to the control gene *actin*, as in Freiman *et al.* (2015), and analysed using the Δ*C*_t_ method (Livak and Schmittgen, 2001).

## Results

### Effects of exogenous ABA application on physiological and metabolic changes during fig fruit ripening

**Fig. 1.**
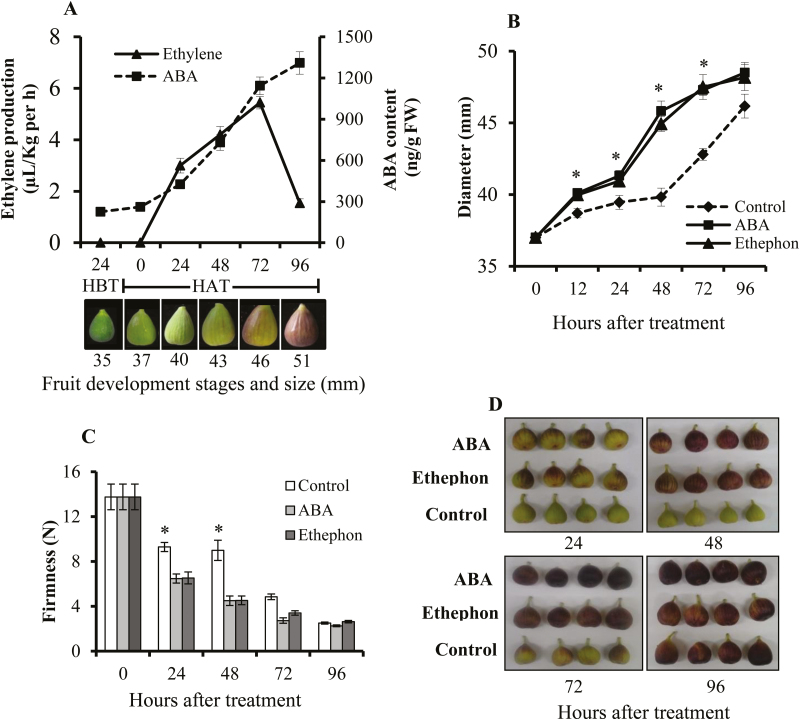
Role of ABA in the fig fruit-ripening process. (A) Changes in endogenous ABA content and ethylene production during the onset of ripening. The fruit developmental stages are distinguished by the diameter and ostiole color. HBT, hours before treatment; HAT hours after treatment. Effects of exogenous ABA application on (B) fruit diameter and (C) firmness. (D) Images showing the different fruit colors and ripening stages at different times after treatment. Fruit treated with ethephon were used as a positive control. Data in (A–C) are means (±SE) of 9 fruit. Significant differences compared with the control were determined using Student’s *t*-test: *P<0.05.

Fig fruit of 37 mm diameter with a greenish-yellow ostiole color were identified as being just before the rapid increase in endogenous ABA and initiation of ethylene production (Fig. 1A). Application of exogenous ABA or ethephon resulted in fruit with significantly larger diameters at 12, 24, 48, and 72 HAT compared to controls (Fig. 1B). ABA- and ethephon-treated fruit were significantly softer at 24 and 48 HAT compared to controls (Fig. 1C), and the change in fruit color associated with ripening started earlier than in controls (Fig. 1D).

### Effects of exogenous ABA application on expression levels of genes of the ABA metabolism and ethylene biosynthesis pathways

**Fig. 2.**
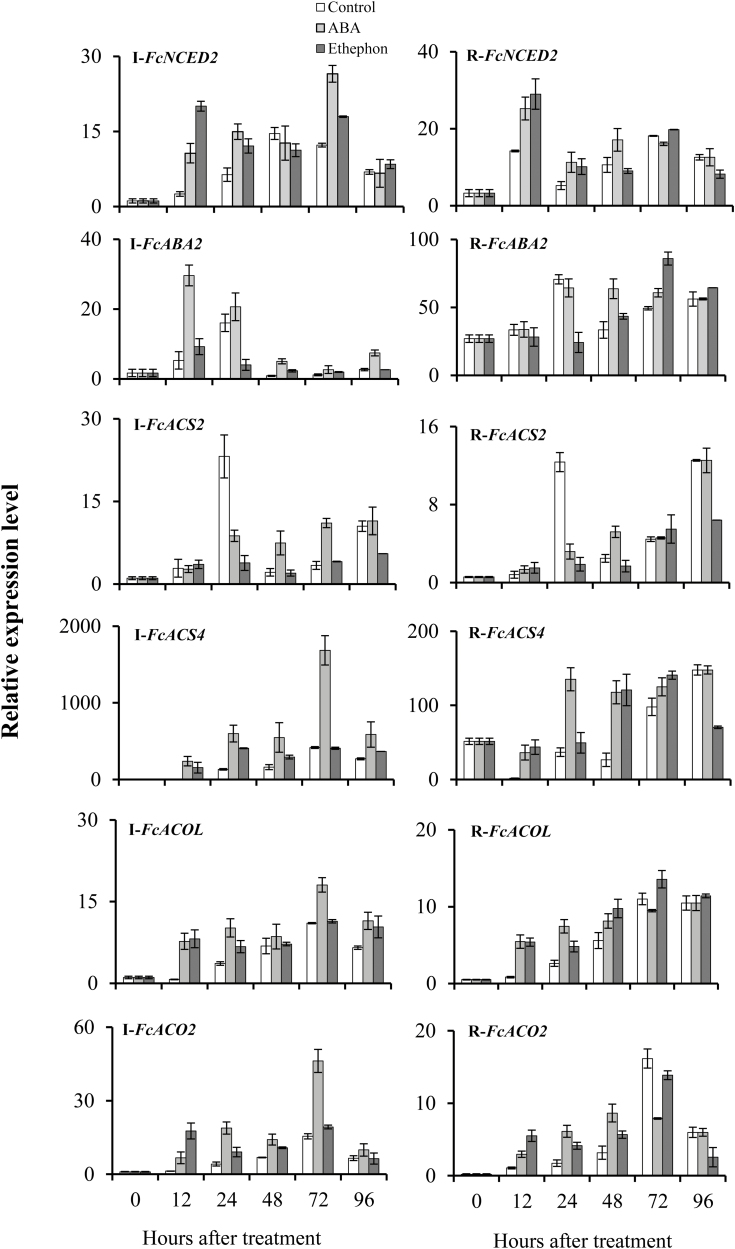
Expression patterns of ABA-biosynthesis genes (*FcNCED2* and *FcABA2*) and ethylene-biosynthesis genes (*FcACS2*, *FcACS4*, *FcACOL*, and *FcACO2*) in ABA- and ethephon-treated fig fruit. I, inflorescence; R, receptacle. Expression is relative to the *actin* gene. Treatment was applied before the onset of ripening. Data are means (±SE) of three biological replicates.

To understand the role of exogenous ABA in fig fruit ripening, the expression levels of six previously characterized (Rosianski *et al.*, 2016*a*) ABA-biosynthesis and catabolism genes were determined (Fig. 2, Supplementary Fig. S1). Exogenous ABA application during the onset of ripening increased the expression levels of these genes. In particular, expression of *FcNCED2*, the key ABA-biosynthesis gene in fig fruit, was increased by ABA and ethephon treatments in both the inflorescences (4.2- and 7.9-fold change, respectively) and the receptacles (1.8- and 2.1-fold change, respectively) at 12 HAT, as compared to untreated controls (Fig. 2). *FcNCED2* continued to be expressed at moderately high levels in ABA- and ethephon-treated fruit in the inflorescences (2.4- and 1.9-fold change, respectively) and the receptacles (2.2- and 1.9-fold change, respectively) at 24 HAT. In addition, moderately high expression of *FcNCED2* was maintained at 72 HAT in inflorescences, but not in receptacles. ABA treatment also increased the expression of the ABA-biosynthesis gene *FcABA2*, to 5.6-fold that of controls in the inflorescence at 12 HAT (Fig. 2). Unexpectedly, ethephon down-regulated *FcABA2* expression at 24 HAT in the inflorescence (Fig. 2).

We then determined the effect of exogenous ABA application on ethylene-biosynthesis genes in the fruit (Fig. 2, Supplementary Fig. S1). *FcACS4* was highly up-regulated by ABA and ethephon in the inflorescences (85.4- and 55.3-fold change, respectively) and in the receptacles (22.2- and 26.8-fold change, respectively) at 12 HAT, as compared to untreated controls. In addition, it was moderately up-regulated by ABA treatment in the inflorescences at 24, 48, and 72 HAT (4.5-, 3.4-, and 4.1-fold change, respectively). In ethephon-treated inflorescences, *FcACS4* was moderately up-regulated at 24 HAT (3.1-fold change), whilst in the receptacles it was moderately up-regulated by ABA treatment at 24 and 48 HAT (3.7- and 4.4-fold change, respectively). Similarly, *FcACS4* was moderately up-regulated at 48 HAT (4.5-fold change) in ethephon-treated receptacles (Fig. 2). The ethylene-biosynthesis gene *FcACOL* was highly up-regulated at 12 HAT by ABA and ethephon application in both the inflorescences (10.8- and 11.5-fold change, respectively) and the receptacles (6.6- and 6.5-fold change, respectively) compared to controls. At 24 HAT, it was moderately up-regulated by ABA in the inflorescences (2.8-fold change) and the receptacles (2.8-fold change). Similarly, compared to controls, *FcACO2* was up-regulated by ABA at 12 and 24 HAT in both the inflorescences (5.3- and4.5-fold change, respectively) and the receptacles (2.8- and5.2-fold change, respectively), and it was also up-regulated by ethephon at 12 and 24 HAT in the inflorescences (13.9- and 2.2-fold change, respectively) and the receptacles (3.5- and 2.4-fold change, respectively) (Fig. 2). Interestingly, in the ABA-treated receptacles, *FcSAM2* and *FcSAM3* were moderately up-regulated (2.4- and 2.9-fold change, respectively) at 12 HAT (Supplementary Fig. S1).

### Effects of fluridone and NDGA application on physiological and metabolic alterations during fig fruit ripening

**Fig. 3.**
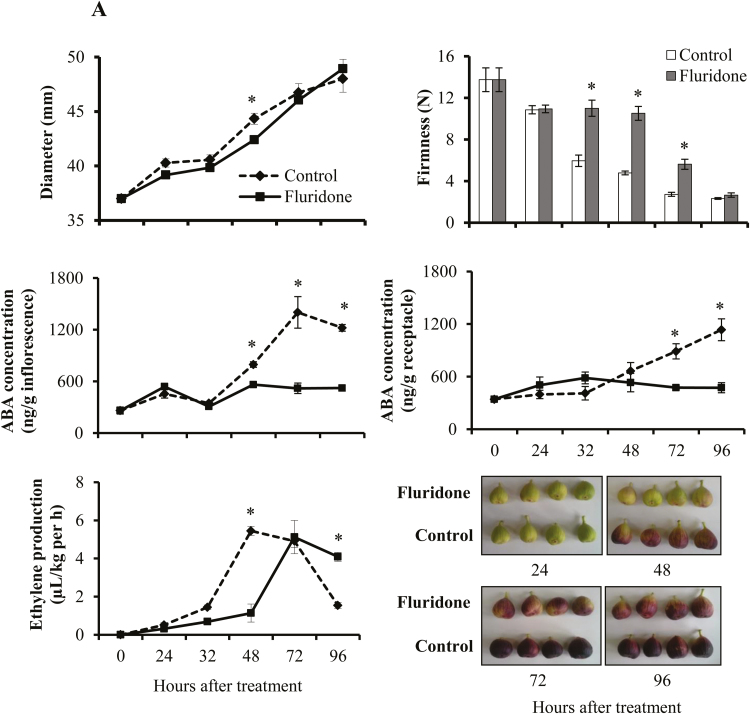

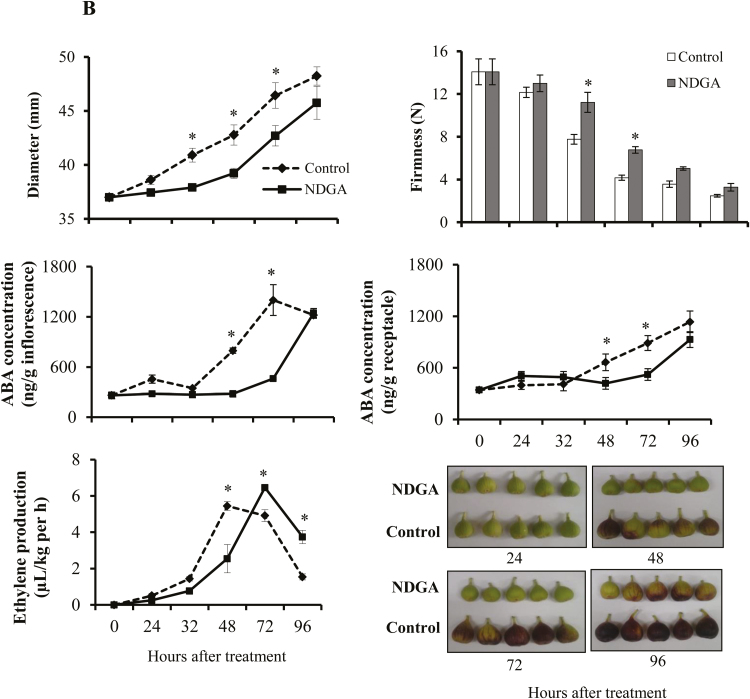
Effects of (A) fluridone and (B) NDGA on diameter, firmness, ABA production in inflorescences and receptacles, and changes in color of fig fruit. Fluridone and NDGA were applied before the onset of ripening. Data are means (±SE) of 9 fruit. Significant differences compared with the control were determined using Student’s *t*-test: *P<0.05.

Fluridone treatment delayed the ripening of treated fig fruit; the fruit diameter was significantly smaller at 48 HAT and fruit remained significantly firmer at 32, 48, and 72 HAT compared to controls (Fig. 3A). The effect of fluridone on fruit firmness appeared to be transient as control and treated fruit showed no significant difference at 96 HAT. In addition, by 96 HAT ABA levels had increased 6- and 4-fold in the inflorescences and receptacles, respectively, of untreated fruit. Notably, we found that fluridone prevented ABA accumulation in both the inflorescences and receptacles, and delayed ethylene production.

Similarly, NDGA also delayed ripening, with treated fruit having significantly smaller diameters and remaining firmer at 32, 48, and 72 HAT compared to controls (Fig. 3B). NDGA significantly delayed ABA accumulation as well as ethylene production in the inflorescences and receptacles.

### Effects of fluridone and NDGA on expression levels of ABA metabolic pathway and ethylene-biosynthesis genes

**Fig. 4.**
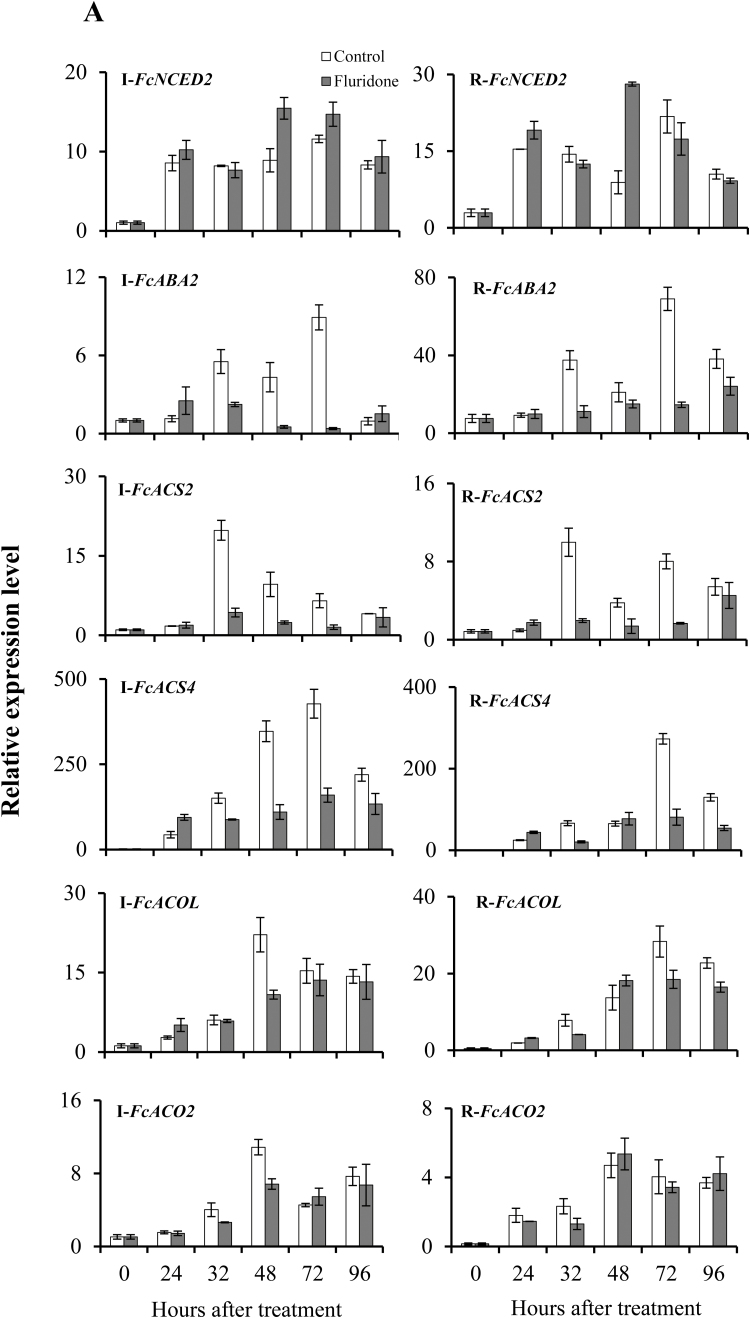

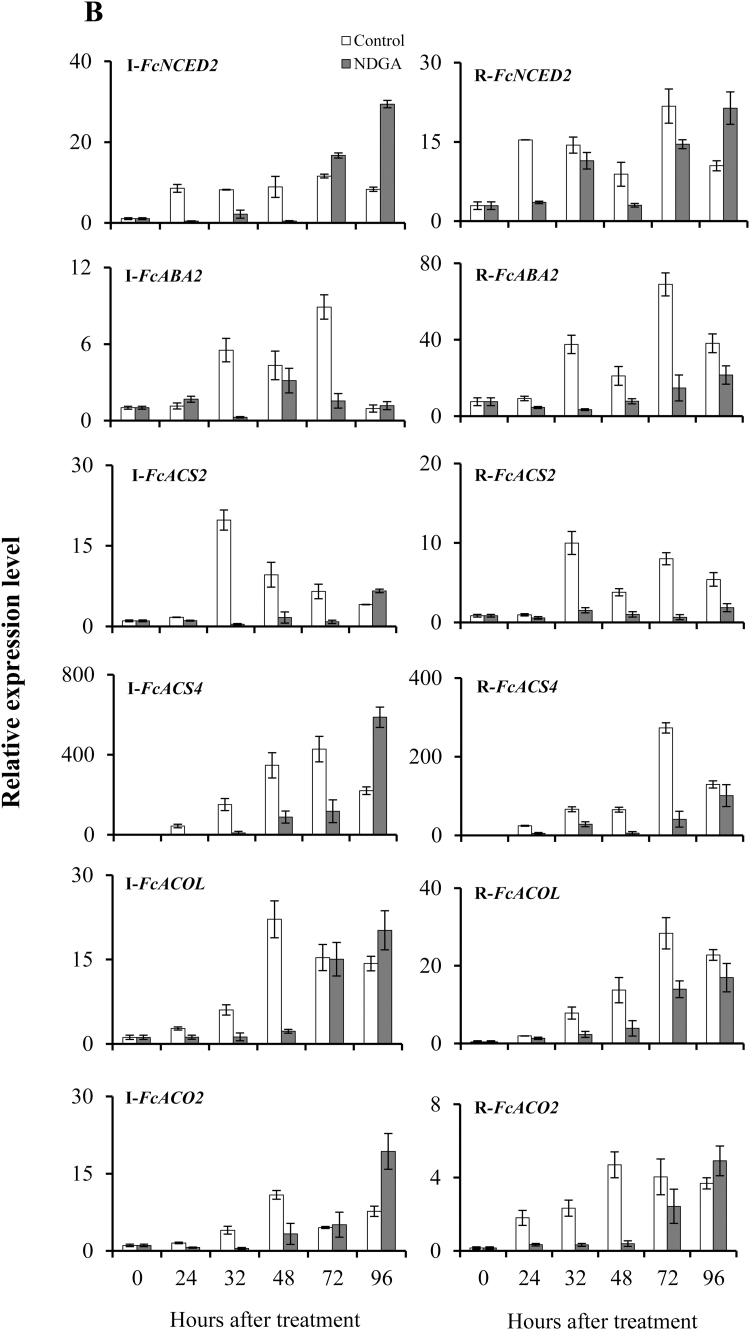
Expression patterns of ABA-biosynthesis genes (*FcNCED2* and *FcAB*A*2*) and ethylene-biosynthesis genes (*FcACS2*, *FcACS4*, *FcACOL*, and *FcACO2*) in (A) fluridone-treated and (B) NDGA-treated fig fruit. I, inflorescence; R, receptacle. Expression is relative to the *actin* gene. Treatment was applied before the onset of ripening. Data are means (±SE) of three biological replicates.

To investigate the changes in expression patterns of ripening-related genes caused by fluridone and NDGA, the expression of genes of the ABA biosynthetic and catabolic pathways and ethylene biosynthesis were examined (Fig. 4, Supplementary Fig. S2). Fluridone did not significantly down-regulate *FcNCED2* (Fig. 4A); however, compared to controls, it inhibited the expression of *FcABA2* in inflorescences at 32, 48, and 72 HAT (2.5-, 8.4-, and 22.6-fold change, respectively) and in receptacles at 32 and 72 HAT (3.4- and 4.7-fold change, respectively). The ethylene-biosynthesis gene *FcACS2* was down-regulated at 32, 48, and 72 HAT in both inflorescences (4.6-, 4-, and 4.3-fold change, respectively) and receptacles (5.1-, 2.7-, and 4.8-fold change, respectively) compared to controls. In addition, *FcACS4* was down-regulated at 48 and 72 HAT in inflorescences (3.2- and 2.7-fold change, respectively) and at 32 and 72 HAT in receptacles (3.3- and 3.7-fold change, respectively). Similarly, expression levels of *FcACOL* and *FcACO2* were slightly reduced at 48 HAT in inflorescences as compared to controls.

Interestingly, NDGA treatment caused down-regulation of *FcNCED1* at 32, 48, and 72 HAT in the receptacles (3.6-, 7.1-, and 9.8-fold change, respectively; Supplementary Fig. S2B). In addition, *FcNCED2* was significantly inhibited at 24, 32, and 48 HAT in inflorescences (18.4-, 3.8-, and 20-fold change, respectively) and in receptacles at 24 and 48 HAT (4.3- and 2.9-fold change, respectively; Fig. 4B). At 32 and 72 HAT, *FcABA2* was inhibited in both inflorescences and receptacles. Expression of the ABA-catabolism gene *FcABA8OX* was elevated at 48, 72, and 96 HAT in both NDGA-treated inflorescences (13-, 5-, and 2.4-fold change, respectively) and receptacles (4.3-, 3.5-, and 3.2-fold change, respectively) compared to controls (Supplementary Fig. S2B). The genes responsible for the first steps of ethylene biosynthesis, *FcSAM2* and *FcSAM3*, were moderately down-regulated in inflorescences (2.8- and 2.4-fold change, respectively) and receptacles (2.9- and 2.2-fold change, respectively) at 12 HAT compared to controls (Supplementary Fig. S2B). In addition, *FcACS2* and *FcACS4* were down-regulated in inflorescences and receptacles of NDGA-treated fruit up to 72 HAT. In particular, *FcACS2* was strongly down-regulated at 32 HAT (47.2-fold change) in inflorescences (Fig. 4B). Similarly, *FcACS4* was strongly down-regulated by NDGA treatment at 24 and 32 HAT (24.7- and 16.5-fold change, respectively) in inflorescences. NDGA-treated fruit also showed strong down-regulation of *FcACOL* at 48 HAT (9.9-fold change) and *FcACO2* at 32 HAT (8.6-fold change) in the inflorescences.

### Effects of exogenous ABA application on expression levels of MADS-box, NAC, and ERF genes

**Fig. 5.**
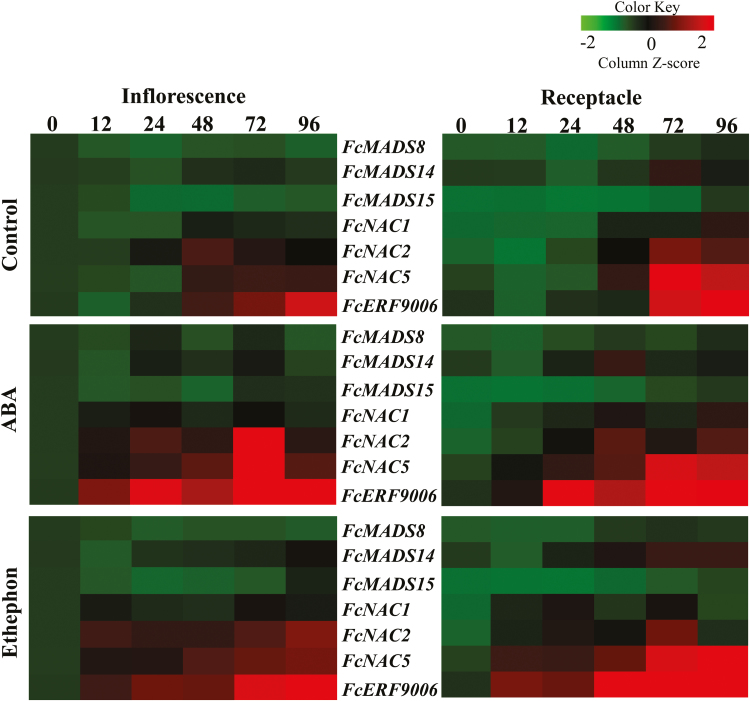
Expression patterns of MADS-box, NAC, and ERF genes in inflorescences and receptacles of fig fruit following on-tree exogenous ABA and ethephon application before the onset of ripening. The numbers at the top indicate the hours after treatment.

To investigate the effects of ABA application on expression of potential ripening-regulator and ethylene signal-transduction genes, 15 MADS-box, 10 NAC, and 12 ERF genes were analysed (Fig. 5, Supplementary Figs. S3, S5, S7). *FcMADS8*, *FcMADS14*, and *FcMADS15* were moderately up-regulated relative to controls by treatment with ABA and ethephon in inflorescences at 24 HAT (Fig. 5). Similarly, *FcMADS8* and *FcMADS14* were up-regulated in receptacles at 24 and 48 HAT, while *FcMADS15* was up-regulated at 48 and 72 HAT. *FcNAC1*, *FcNAC2*, and *FcNAC5* were up-regulated by ABA and ethephon treatment at 12 and 24 HAT in both inflorescences and receptacles, and *FcERF9006* was up-regulated at 12, 24, and 48 HAT in both inflorescences and receptacles.

### Effects of fluridone and NDGA on expression levels of MADS-box, NAC, and ERF genes

**Fig. 6.**
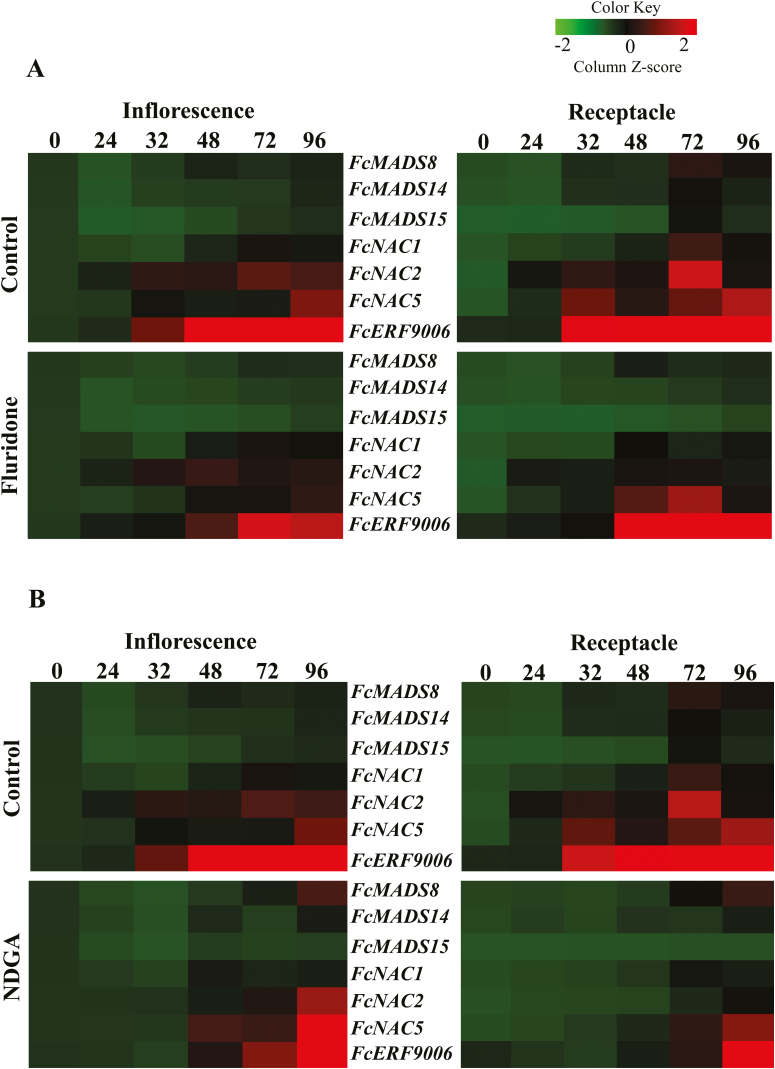
Expression patterns of MADS-box, NAC, and ERF genes in inflorescences and receptacles of fig fruit treated with (A) fluridone and (B) NDGA. The numbers indicate the hours after treatment.

To determine the effects of fluridone and NDGA on the expression of potential ripening-regulator and ethylene signal-transduction genes, MADS-box, NAC and ERF genes were analysed (Fig. 6, Supplementary Figs. S4, S6, S8). Compared to controls, *FcMADS8*, *FcMADS14*, and *FcMADS15* were moderately down-regulated by fluridone and NDGA treatments at 32 HAT in inflorescences and receptacles (Fig. 6). Similarly, *FcNAC1* was slightly down-regulated by NDGA at 72 HAT in inflorescences, and also at 48 and 72 HAT in receptacles (Fig. 6B). There was no effect of fluridone on *FcNAC1* in inflorescences, although this gene was down-regulated in receptacles at 72 HAT (Fig. 6A). Interestingly, *FcNAC2* and *FcNAC5* were down-regulated by NDGA at 32 HAT in both inflorescences and receptacles (Fig. 6B). In addition, *FcNAC5* was down-regulated by fluridone at 32 HAT in inflorescences and receptacles (Fig. 6A). *FcERF9006* was down-regulated by both fluridone and NDGA up to 72 HAT in both inflorescences and receptacles (Fig. 6).

### Correlation between ripening-related genes and ABA in fluridone- and NDGA-treated fruit

**Fig. 7.**
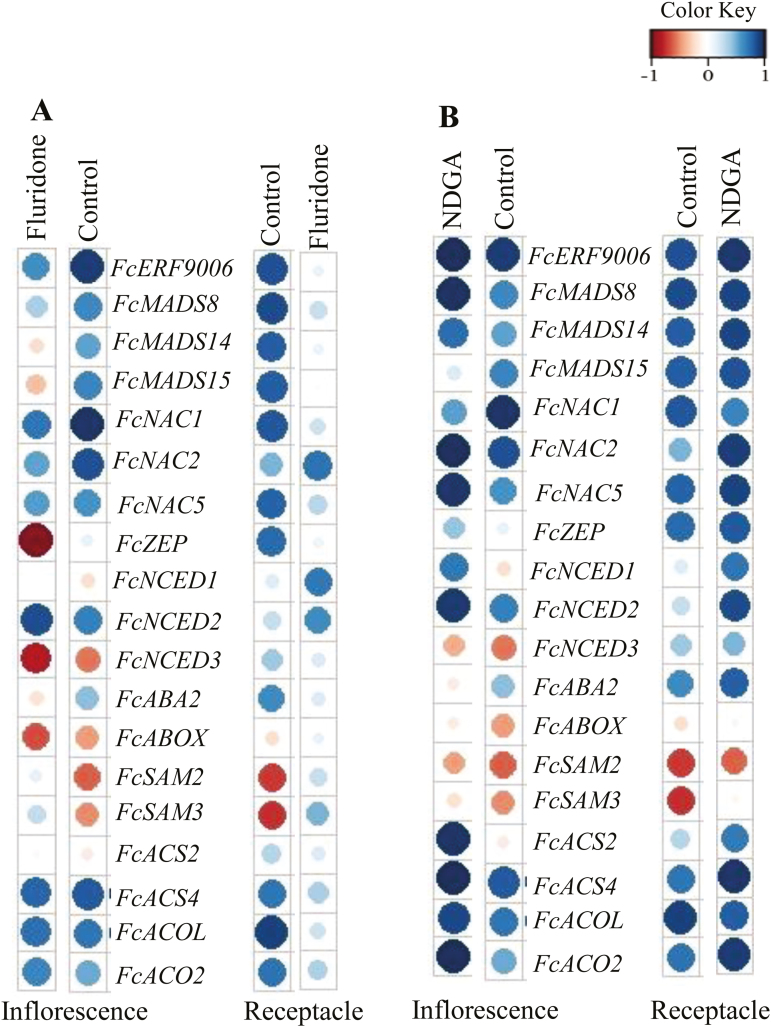
Graphical representation of the correlation matrix between genes and ABA during ripening in inflorescences and receptacles following (A) fluridone and (B) NDGA treatment. Pearson’s correlations were applied using the corrplot package. The color intensity and the size of the circle are proportional to the correlation coefficients. Positive and negative correlations are presented from blue to red, respectively (see key).

Potential coordination between expression of ripening-related genes and ABA during fig fruit ripening was determined by Pearson’s correlation analysis. The analysis reflected the degree of coordination between gene expression and hormonal changes in the reproductive (inflorescence) and non-reproductive (receptacle) tissues of treated and non-treated fruit (Fig. 7). Fluridone treatment strongly altered the correlation between ABA and ripening-related genes in both tissues. In particular, *FcSAM2* and *FcSAM3* were negatively correlated with ABA in untreated fruit, whereas they were positively correlated in inflorescences and receptacles of fluridone-treated fruit (Fig. 7A). Fluridone negatively altered the correlation of *FcABA2* with ABA in the inflorescence but not in the receptacle. In addition, *FcZEP*, *FcMADS14*, and *FcMADS15* were negatively correlated with ABA in the inflorescence, whereas there was no correlation in the receptacle (Fig. 7A).

Interestingly, NDGA treatment resulted in a positive correlation of *FcACS2* and *FcNCED1* with ABA in the inflorescence; however, *FcABA2* was negatively correlated with ABA in the NDGA-treated inflorescence (Fig. 7B). *FcNCED3* was negatively correlated with ABA in inflorescences but positively correlated with ABA in the receptacles of all treated and untreated fruit. Furthermore, highly positive correlations of the key ABA-biosynthesis gene *FcNCED2* and of the ethylene-biosynthesis genes *FcACS4*, *FcACOL*, and *FcACO2* with ABA were detected in the inflorescences of treated and untreated fruit (Fig. 7).

## Discussion

Fig fruit has traditionally been perceived as climacteric because its ripening process follows an increase in respiration rate and ethylene production (Chessa *et al.*, 1992). However, ripening-related ethylene production has been found to increase in an unexpected auto-inhibitory manner after application of 1-MCP (Sozzi *et al.*, 2005; Owino *et al.*, 2006; Freiman *et al.*, 2012). This paradox was explained by Freiman *et al.* (2015) in a molecular study of potential ripening-regulatory and ethylene-related genes in the receptacles (vegetative tissue) and inflorescences (reproductive tissue). More recently, endogenous ABA production has been observed to increase as the result of an increase in expression levels of ABA-biosynthesis genes at the onset of fruit ripening (Rosianski *et al.*, 2016*a*). These data suggest that both ABA and ethylene are crucial players in the ripening process of figs, and we examined their potential interplay in the current study.

### Exogenous ABA enhances fruit ripening by inducing the expression of genes in the ABA metabolism and ethylene biosynthesis pathways

Exogenous ABA treatment induced earlier onset of ripening, followed by a rapid increase in fruit diameter, softening, and purple coloration of the outer peel. Ethephon had the same effect and was used as a positive control (Fig. 1). Concomitant with the early onset of ripening, both ABA-biosynthesis genes (particularly *FcNCED2*) and ethylene-biosynthesis genes (particularly *FcACS4*, *FcACOL*, and *FcACO2*) were up-regulated as a result of exogenous ABA treatment in inflorescences and receptacles at 12 and 24 HAT (Fig. 2). These results are in general agreement with previous work on tomato where exogenous ABA was found to promote ripening by enhancing ethylene biosynthesis (Zhang *et al.*, 2009*b*; Mou *et al.*, 2016). An increase in endogenous autocatalytic ethylene production as a result of application of exogenous ABA is well known in several other fruits such as banana (Jiang *et al.*, 2000), melon (Sun *et al.*, 2013), peach, and grapes (Zhang *et al.*, 2009*a*). Exogenous ABA also enhances the ripening process of non-climacteric strawberry fruit (Jia *et al.*, 2011). In citrus, exogenous ABA accelerates ripening and induces expression of the ethylene-biosynthesis gene *CsACO1*. It also enhances fruit color, significantly decreases organic acid content, and increases sugar accumulation (Wang *et al.*, 2016).

The key ABA-biosynthesis gene *NCED* has been cloned and characterized from various climacteric fruit species, such as apple (Lara and Vendrell, 2000), peach (Soto *et al.*, 2013), tomato (Burbidge *et al.*, 1999), and melon (Sun *et al.*, 2013), as well as from non-climacteric fruit such as orange (Rodrigo *et al.*, 2006), grape (Wheeler *et al.*, 2009), and strawberry (Jia *et al.*, 2011). In particular, in climacteric tomato fruit, *LeNCED1* initiates ABA biosynthesis at the onset of fruit ripening, and may act as a ripening inducer (Zhang *et al.*, 2009*b*). Similarly, *FaNCED1* and *FaNCED2* have been reported to regulate ABA biosynthesis during the onset of ripening in non-climacteric strawberry fruit (Jia *et al.*, 2011; Ji *et al.*, 2012). To date, three genes encoding the NCED enzyme have been isolated from the inflorescence and receptacle tissues of both pollinated and parthenocarpic fig fruit during ripening (Rosianski *et al.*, 2016*a*). Our current study showed that expression of *FcNCED2* was up-regulated as early as 12 HAT, while expression of the ethylene-biosynthesis gene *FcACS4* was only up-regulated at 24 HAT in control fruit. In addition, another ABA-biosynthesis gene, *FcABA2*, showed a similar temporal pattern of up-regulation, but only in control fruit inflorescences (Fig. 2). The simultaneous up-regulation of key ABA- and ethylene-biosynthesis genes, especially in the inflorescence, suggested that there was major up-regulation of ethylene-biosynthesis genes after the up-regulation of ABA-biosynthesis genes, as seen in tomato (Zhang *et al.*, 2009*b*; Mou *et al.*, 2016), and confirmed that the ethylene-dependent ripening process in fig is coordinated by the reproductive part of the fruit—the inflorescence—as stated by Freiman *et al.* (2015) and Rosianski *et al.* (2016*a*).

### Fluridone and NDGA reduce expression of ABA-biosynthesis genes, thereby altering fruit ripening

Fruit treated with fluridone showed delayed onset of ripening, followed by a slower increase in diameter and a slower decrease in firmness of the fruit compared with controls (Fig. 3A). Endogenous ABA accumulation and ethylene production were strongly suppressed as a result of the down-regulation of ABA- and ethylene-biosynthesis genes. Similarly, application of NDGA reduced the expression of ABA-biosynthesis genes. Specifically, NDGA reduced the expression of both *FcNCED2* and *FcABA2*, whereas fluridone only lowered the expression level of *FcABA2* (Fig. 4). Although fluridone and NDGA act in different ways to suppress ABA biosynthesis, the ethylene-biosynthesis genes *FcACS2*, *4*, *FcACOL*, and *FcACO2* were strongly down-regulated by both inhibitors, as also occurs in the fruit of tomato (Zhang *et al.*, 2009*b*) and strawberry (Jia *et al.*, 2011). In tomato, down-regulation of *SlNCED1* by RNAi leads to down-regulation of genes encoding major cell wall-catabolic enzymes during ripening, resulting in firmer fruit (Sun *et al.*, 2012), whilst in strawberry, down-regulation of *FaNCED1* results in a significant decrease in ABA levels and in fruit that lack color (Jia *et al.*, 2011). As with *SlNCED1* in tomato (Sun *et al.*, 2012), *FcNCED2* in fig was down-regulated by NDGA (*FcNCED2* shares 71.72% identity with *SlNCED1*, Supplementary Fig. S9); it is therefore plausible that NDGA treatment altered the expression of genes related to regulation of cell wall modification in fig: further experiments are needed to clarify this point. Application of NDGA to fig up-regulated the ABA-catabolism pathway gene *FcABA8OX*, which could have been responsible for the decrease in total active ABA accumulation. This would be in agreement with work in tomato by Mou *et al.* (2016), where *ABA8OX* was slightly up-regulated after NDGA treatment; however, no effect of fluridone was observed.

### Responses of potential ripening regulators to exogenous ABA, fluridone, and NDGA treatments

The ripening process in fleshy fruits is regulated by plant hormones and involves numerous transcription factors (Adams-Phillips *et al.*, 2004; Karlova *et al.*, 2014). Members of the MADS-box gene family have been found to regulate ripening in several climacteric species: *RIN* and *TAGL1* in tomato, *PLENA* in *Prunus persica* (peach), *MADS1–5* in banana, and *MADS8* and *MADS9* in apple (Vrebalov *et al.*, 2002, 2009; Itkin *et al.*, 2009; Tadiello *et al.*, 2009; Elitzur *et al.*, 2010; Ireland *et al.*, 2013). In addition, genes from the MADS-box gene family are involved in non-climacteric fruit ripening, such as *MADS9* in strawberry (Seymour *et al.*, 2011). In the unique climacteric fig fruit, eight MADS-box transcripts have been identified and partially isolated from fruit developing on the tree (Freiman *et al.*, 2014). An in-depth study of the transcriptome data of fig fruit during different ripening stages revealed seven new MADS-box transcripts (Rosianski *et al.*, 2016*a*). In addition, *FcMADS8*, which is highly similar to *SlRIN*, shows an increase in expression during the ripening of both fig inflorescences and receptacles (Freiman *et al.*, 2015). Here, we found that *FcMADS8*, *14*, and *15* were moderately up-regulated by ABA application in both inflorescences and receptacles at 24 HAT, whereas they were inhibited by fluridone and NDGA (Figs 5, 6). This was similar to tomato fruit, where expression of *MADS-RIN* is elevated by exogenous ABA, and suppressed by NDGA when endogenous ABA is inhibited (Mou *et al.*, 2016).

Among the 27 NAC transcripts of fig fruit described by Freiman *et al.* (2014), *FcNAC1*, *2*, and *5* showed moderate up-regulation by ABA treatment (Fig. 5). On the other hand, *FcNAC2* and *5* were down-regulated by both fluridone and NDGA treatments while *FcNAC1* was down-regulated by NDGA only in the receptacle (Fig. 6). A similar effect has been noted in tomato fruit where *NOR*, a member of the NAC domain family that functions upstream of ethylene in the tomato fruit ripening cascade, is elevated by exogenous ABA and suppressed by NDGA (Mou *et al.*, 2016). In this context, our results suggested that *FcNAC1*, *2*, and *5* interacted in their role in fig fruit ripening, similar to *MaNAC1* and *MaNAC2* in banana fruit ripening via interaction with the ethylene-signaling pathway (Shan *et al.*, 2012). Furthermore, overexpression of *SlNAC1* has been shown to regulate tomato fruit ripening through both ethylene-dependent and ABA-dependent pathways (Ma *et al.*, 2014). Reduced expression of *SlNAC4* by RNAi in tomato results in delayed fruit ripening, suppression of chlorophyll breakdown, a decrease in ethylene biosynthesis by a reduction in the expression of ethylene-biosynthesis genes, and a reduction in carotenoids by alterations in fluxes in the carotenoid pathway (Zhu *et al.*, 2014). Surprisingly, Kou *et al.* (2014) showed that *SlNAC4* is down-regulated by ABA treatment whereas *SlNAC5*, *6*, *7*, and *9* are up-regulated in tomato fruit.

The ERFs are a large family of transcription factors that includes gene repressors and activators, with a degree of functional redundancy among its members (Klee and Giovannoni, 2011; Licausi *et al.*, 2013). In fig fruit, ERFs are positive ripening regulators, except for *FcERF12185* that is only highly expressed at the fully ripe stage and is probably not responsible for the climacteric rise in ethylene or the metabolic ripening processes in the earlier stages (Freiman *et al.*, 2015). Among the ERF transcripts isolated by Freiman *et al*. (2015), we demonstrated here that *FcERF9006* expression is up-regulated by exogenous ABA and ethephon treatment, whereas it is down-regulated by fluridone and NDGA in both inflorescences and receptacles (Figs 5, 6). In climacteric tomato, *LeERF2*, *LeERF3*, and *LeERF4* are down-regulated by ABA treatment and *LeERF4* is more highly expressed after NDGA treatment (Mou *et al.*, 2016). Indeed, temporary down-regulation of most *FcERF*s was observed in the unique climacteric fig fruit following pre-harvest treatment with 1-MCP (Freiman *et al.*, 2015).

### Non-climacteric characteristics of fig fruit exclude them from the conventional climacteric category

Despite the classification of fig fruit as climacteric, when harvested prior to complete ripening they never reach the commercially desirable parameters of size, color, flavor, and texture (Flaishman *et al.*, 2008). Surprisingly, 1-MCP markedly enhances ethylene production in figs (Sozzi *et al.*, 2005; Owino *et al.*, 2006; Freiman *et al.*, 2012), similar to its effect in non-climacteric fruit such as citrus (McCollum and Maul, 2007). This confirms the non-climacteric, auto-inhibitory regulation of ethylene synthesis, rather than the autocatalytic pattern typical of climacteric fruit (Freiman *et al.*, 2012). Apart from ethylene, several recent studies have shown that ABA plays an important role in the regulation of fruit development and ripening in both climacteric and non-climacteric fruit (Zhang et al., 2009*a*, 2009*b*; Sun *et al.*, 2012; Leng *et al.*, 2014). In the mature fig fruit, endogenous ABA is present before ethylene is produced, and is supposed to trigger the ripening process (Fig. 1A). The increase in accumulation of endogenous ABA during the onset of fig ripening is similar to that in other climacteric fruit such as tomato, avocado, and apple (Chernys and Zeevaart, 2000; Lara and Vendrell, 2000; Srivastava and Handa, 2005; Zhang *et al.*, 2009*b*; Leng *et al.*, 2014). However, instead of tapering off after the onset of ripening, as in climacteric fruit, the endogenous ABA content in control plants continued to rise until the fig fruit was fully ripe, as in non-climacteric strawberry fruit (Fig. 1A; Jia *et al.*, 2011). Interestingly, this phenomenon was observed in both the reproductive inflorescence tissue and the vegetative receptacle tissue, with high expression of major ABA-biosynthesis pathway genes (Figs 2, 4). On the other hand, expression of ethylene-related genes was different for the two tissues, with only the inflorescence showing expression similar to that in climacteric fruit (Freiman *et al.*, 2015). In agreement with previous studies, our results showed that ethylene was produced at high levels at the 30–50% ripening stage, with significantly high expression of the ethylene-biosynthesis gene *FcACS4* in the inflorescence, although expression levels rose in both tissues (Freiman *et al.*, 2012, 2015; Fig. 1A). Increased expression of ethylene-biosynthesis genes was also observed in petioles (vegetative tissues) of fruit at different ripening stages—from green to fully ripe (Supplementary Fig. S10). These expression levels were very low compared to those in the inflorescences and receptacles, and they played no part in fruit ripening. Similarly, very low expression of ethylene-biosynthesis genes in receptacles relative to inflorescences would lead to the production of minor amounts of ethylene, which might not be enough for the ripening process, but a high amount of endogenous ABA production until full ripeness, as in strawberry, could lead to non-climacteric ripening by promoting sugar accumulation, fruit pigmentation, and softening, as described in a non-climacteric fruit-ripening model (Li *et al.*, 2011). This distinct mechanism in the receptacle could be synchronized by the ABA-biosynthesis genes *FcZEP*, *FcNCED1*, and *FcNCED3*, the expression of which was positively correlated with ABA in the receptacle but negatively correlated in the inflorescence during natural ripening of untreated fruit (Fig. 7). Different ripening mechanisms for specific organs have also been reported in strawberry, where ethylene is involved in the ripening of achenes (reproductive organ) but not the receptacles (vegetative organ) (Merchante *et al.*, 2013). Indeed, strawberry is considered a non-climactic fruit where ABA promotes ripening by increasing its endogenous levels until full ripeness (Jia *et al.*, 2011). This unique characteristic of ripening, showing both climacteric and non-climacteric behavior, has also been reported in ‘Elizabeth’ melon (*Cucumis melo*), although it is regarded as a climacteric fruit (Sun *et al.*, 2013).

Non-climacteric fruit are considered to be a separate group that does not follow the typical climacteric ripening pattern. However, the identification of MADS-box genes in both climacteric and non-climacteric fruit suggests that at least some aspects of ripening are shared between these two categories (Daminato *et al.*, 2013). In strawberry, a MADS-box *SEPALLATA* gene (*SEP1/2*) is needed for normal fruit development and ripening (Seymour *et al.*, 2011). Similarly, in banana, which is classified as a climacteric fruit, the MADS-box *SEP3* gene also displays ripening-related expression (Elitzur *et al.*, 2010). In the fig fruit, *FcMADS8* expression increases during ripening on the tree and is inhibited by 1-MCP treatment (Freiman *et al.*, 2015). Interestingly, *FcMADS8* was inhibited by fluridone and NDGA in the inflorescence at 32 and 48 HAT (Fig. 6). During the ripening of pepper fruit, genes involved in ethylene biosynthesis are not induced; however, genes downstream of ethylene perception, such as cell wall-related genes, *ERF3*, and carotenoid-biosynthesis genes, are up-regulated (Osorio *et al.*, 2012). In fig fruit, *FcERF12185* is up-regulated by 1-MCP treatment during ripening and may play a role in regulating ethylene-synthesis system 1 and in causing the non-climacteric behavior of ethylene production (Freiman *et al.*, 2015). However, expression of *FcERF12185* was not affected by application of ABA, fluridone, or NDGA in our current study (Supplementary Figs S7, S8). In contrast, the expression level of *FcERF9006* was altered by application of ABA, fluridone, and NDGA, which suggests a role for this gene in non-climacteric and/or ABA-dependent ripening of fig fruit (Figs 5, 6).

**Fig. 8.**
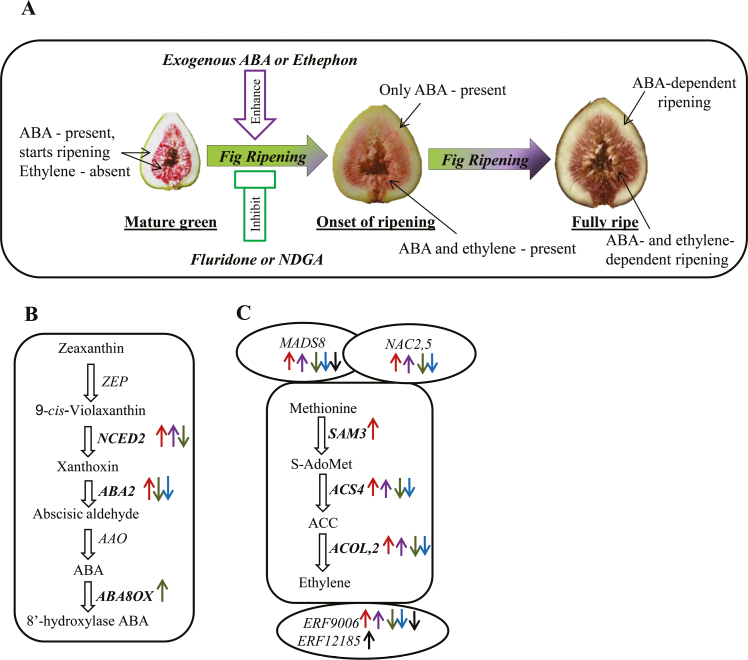
Proposed model of ABA-regulatory and -biosynthesis genes in fig fruit. (A) ABA interplay with ethylene in fig fruit during ripening. (B) Changes in activity of ABA metabolic pathway genes in the fruit following on-tree exogenous application of ABA, ethephon, NDGA and fluridone. (C) Changes in activity of ethylene-biosynthesis, MADS-box, NAC, and ERF gene in the fruit following on-tree exogenous application of ABA, ethephon, NDGA, fluridone, and 1-MCP (Freiman *et al.*, 2015). Downward arrows indicate down-regulated genes in treated fruit compared to untreated controls; upward arrows indicate up-regulated genes. The arrows are color-coded as follows: red, ABA; purple, ethephon; green, NDGA; blue, fluridone; black, 1MCP.

In conclusion, involvement of ABA during fig fruit ripening was confirmed in our study by observed alterations in ripening as the result of the application of exogenous ABA, ethephon, fluridone, and NDGA. The unique ripening nature of fig fruit, in which two tissues act separately, is illustrated in a proposed model (Fig. 8A). The alterations in the expression levels of ABA-metabolic pathway, ethylene-biosynthesis, MADS-box, NAC, and ERF genes in the fruit by on-tree application of ABA, ethephon, fluridone, NDGA, and 1-MCP (Freiman *et al.*, 2015) are summarized in Fig. 8B, C. A highly positive correlation of the ABA-biosynthesis gene *FcNCED2* and the ethylene-biosynthesis gene *FcACS4* with endogenous ABA in the inflorescence demonstrates a key role in fig fruit ripening. We plan to further utilize these as candidate genes for genome editing to slow down the ripening process.

## Supplementary data

Supplementary data are available at *JXB* online.

Table S1. Primers used for high-throughput real-time qPCR.

Fig. S1. Expression pattern of ABA-metabolism and ethylene-biosynthesis genes in ABA- and ethephon-treated fig fruit.

Fig. S2. Expression pattern of ABA-metabolism and ethylene-biosynthesis in fluridone- and NDGA-treated fig fruit.

Fig. S3. Expression pattern of MADS-box genes in inflorescence and receptacle tissues following ABA and ethephon treatment.

Fig. S4. Expression pattern of MADS-box genes in inflorescence and receptacle tissues following fluridone and NDGA application.

Fig. S5. Expression pattern of NAC genes in inflorescence and receptacle tissues following ABA and ethephon application.

Fig. S6. Expression pattern of NAC genes in inflorescence and receptacle tissues following fluridone and NDGA application.

Fig. S7. Expression pattern of ERF genes in inflorescence and receptacle tissues following ABA and ethephon application.

Fig. S8. Expression pattern of ERF genes in inflorescence and receptacle tissues following fluridone and NDGA application.

Fig. S9. Alignment of *FcNCED2* amino acid sequence and *SlNCED1* protein.

Fig. S10. Expression of ethylene-biosynthesis genes in the petiole of fig fruits at different developmental stages during ripening.

Supplementary Material
