# Supplementary material for: The distinct ripening processes in the reproductive and non-reproductive parts of the fig syconium are driven by ABA
Source: J Exp Bot. 2018 Aug 31;70(1):115–31. doi: 10.1093/jxb/ery333 (PMC6305202; doi:10.1093/jxb/ery333)
Supplement: Supplementary Material [file ery333_suppl_supplementary_material.pdf]

Supplementary material for the manuscript:

ABA-driven ripening of fig (*Ficus carica* L.) fruit governs distinct ripening process in reproductive and non-reproductive parts of the syconium

Kumar Lama[1,2], Sharawan Yadav[1], Yogev Rosianski[1], Felix Shaya[1], Amnon Lichter[3], Lijuan Chai[1], Yardena Dahan[1], Zohar Freiman[1], Reut Peer[1] and Moshe A. Flaishman[1*]

[1] Institute of Plant Sciences, Agricultural Research Organization, P.O. Box 6, Bet-Dagan 50250, Israel

[2] The Robert H. Smith Institute of Plant Sciences and Genetics in Agriculture, Faculty of Agriculture, Food and Environment, The Hebrew University of Jerusalem, P.O. Box 12, Rehovot 76100, Israel

[3] Institute of Postharvest and Food Sciences, Agricultural Research Organization, P.O. Box 6, Bet-Dagan 50250, Israel

Emails of all authors: Kumar Lama: kumar.lama@mail.huji.ac.il; Sharawan Yadav: sybu03@gmail.com; Yogev Rosianski: rosyogi1@gmail.com; Felix Shaya: fshaya@agri.gov.il; Amnon Lichter: vtlicht@agri.gov.il; Lijuan Chai: clj2010@cau.edu.cn; Yardena Dahan: yardenad@volcani.agri.gov.il; Zohar Freiman: zohar.freiman@mail.huji.ac.il; Reut Peer: reutp@volcani.agri.gov.il; Moshe A. Flaishman: vhmoshea@agri.gov.il

* To whom correspondence should be addressed. E-mail: vhmoshea@agri.gov.il; Telephone: +97239683394

Table S1. Primers used for high-throughput real-time qPCR.

Gene name	Forward Primer (5'—3')	Reverse Primer (5' —3')
FcZEP	TTCGTGACTGATTTGCGAAG	TTGTGCAACAGTCGGAGAAG
FcNCED1	ATTGCCGAATCTGAGCAAGT	TCTTCTTCCGAACACGATCC
FcNCED2	TACATCATGGCCTTCGTTCA	TGCCTGAAAAGCCAAATCTT
FcNCED3	AGGAGGATGTATGGCACAGG	ACTTCGGCCACAATCTCAAG
FcABA2	TATGTTCAACAACGCCGGTA	TAAGCATGGGAGCCTACACC
FcABA8OX	GAGAGCTTGAGGATGGCAAG	TGCCAAATGGCATGAAAGTA
FcSAM2	ACCTCGACGAGAAAACCATCT	CACCTTAGTTGGGTCCTTTCC
FcSAM3	CCCAAGTAACGGTCGAGTACA	GGGATCACAGGCTTAATGACA
FcACS2	ACGCAGGATTGTTCTTCTGG	GACGGTCTCTTGGTCCATGT
FcACS4	TTCTTGTCACTGCACCGAAC	AGCGACCGAGTTCTCGAATA
FcACOL	GAGCAGGAAACCAAAGTGGT	CCTTCATGGCTTCAAACCTAG
FcACO2	TGCCAAACGCCATAGTGATA	CTTGCCTTATCCTCCACCAA
FcMADS1	GCAGAACGATTGGAAGAACTG	GGTCCATGACAAGAGCAGAAT
FcMADS2	GGAACTGGACCGTGGAGTATT	GCATTGCCTTCTCCTTCTTCT
FcMADS3	GATGACGAGAAAGCTGAAGGAG	TGTAGTAGGCCGAGTTTGAGTTC
FcMADS4	GGCATATGAAGGGAGAAGACC	CTACATCGACTACAATTGCTCCTG
FcMADS5	CGAATGTAGTGGACCCTTGAA	AAGTTGCTGAGCTGCTGAAAG
FcMADS6	TGCTCAGATTCCACTTCGATT	CAACATTCATCCCATCTGGAC
FcMADS7	AGCGCGTTATGAATCCCTACTA	CCTTTGTTTCAGGGTCCTATTG
FcMADS8	TCTTCGTAGGAAGGAAGAATCG	TGCAGAATTAACGCTGTAGCC
FcMADS9	AAGAACTGAAGCAGCTGGAGAC	CCTGTATTGCATTGAGCTCTGA
FcMADS10	TGGGACCTCTAAGTACAAAGGAAC	GTACGGCATAGTTTGCTGATGA
FcMADS11	GGAACAACAGTTGGAAAGGAGT	GTTTGGTGGTAAGGTTTCTCTCC
FcMADS12	ACAGAGAAGCAAGCAAGAAAGG	CCTCCAAGCAATCTGATAAACC
FcMADS13	CAAGGCATTAGAGGAGCAAAAC	GTCGCTTCATCTACTCCATCACT
FcMADS14	CGTCGCTCTTCTTGTCTTCTCTA	CAGTGTGTGTCAGTAAAGTTGCAC
FcMADS15	GGCGAGTGTTTGAATGGTTT	GACCCTCAACTAACCCGTAGTCT
FcNAC1	ACTTCTAGGCCTTGTCCTGTCTC	CCTGACACAGTTGCTCTATTCG
FcNAC2	GAGGACAAACAGAGCAACAGAAG	GGGGAGATTATGGATGGAGAA
FcNAC3	AGGGTAGCCTAGAGAAGAACTACCA	CTCCATTTGGGTTGACTCTCA
FcNAC4	AGGACCTTGACTACTTTGACGTG	CTGAAGAGGAAGCCTCATTGTT

Gene name	Forward Primer (5'—3')	Reverse Primer (5' —3')
FcNAC5	ACGTTTGGTGAGAAAGAGTGGT	GGAGGTCTTCCCTTGTAGAAAAC
FcNAC6	ATCCCAAACAGCTCTTTCTACG	CGGTGTCTTGAGAGAGACTAACC
FcNAC7	GGATGAAGAGCTTGTAGGGTTCTA	GCTGTTCTTGTACTTTCTCCCTCT
FcNAC8	GAAGAGCTCGTCGTTCACTACC	CCCCATTCGGATACTTCCTATC
FcNAC9	TGATCATACCTTCCCTAACCAGAG	GGATTTGGATCTAGTGCTACACG
FcNAC10	GCAAGCTGAGCTAGGATCTTCTTA	CCACCACTAGAGGCCAGATTATT
FcEIL1	GGAGGGTCCAACCTATGGAT	GTGATGGCTGAGGCAGTGTA
FcEIL2	GGTTGTTATGGGAGGTAATATCC	GTCCGCAATATTGTCATTGG
FcEIL3	CGGTAGTGGCTCCTAAGATTAAA	AATCCTGATAGGGGCATTGA
FcERF12185	GAGTCGATCGGGAGTGAGTT	CTCGCCGTAAAAGTACTCATCA
FcERF9211	ACTAGTTTCGCCGCTAGCAA	ACTAGTTTCGCCGCTAGCAA
FcERF5086	CACCGTCCTACGTTTCCAAT	AACCTCCCAAATCAAATCAGG
FcERF9690	ACGGAGAGAGCACGATGG	CTGAACCGGAATTCGTGACT
FcERF10238	GTTAGATTCCCGTTCCAGCA	GTTGATACGGGACGCTCTTG
FcERF10715	GCTTAGCAGACCGGATTTCA	TTCAACCACGGAAGACGAGT
FcERF9816	CGAACAAGAGGTCGAGGATT	AAGATGAGGTCGGGGAAGTT
FcERF14757	CAGCCCATCAACACGACC	CTAGCCAGAGGCGAGTCCT
FcERF9006	CGGACCTCTACCACTATGCAC	TGTAATGAAAGGGAGCTGCTG

Fig. S1. Expression pattern of ABA-metabolism genes (*FcZEP, FcNCED1, FcNCED3* and *FcABA8OX*) and ethylene biosynthesis genes (*FcSAM2* and *FcSAM3*) in ABA- and ethephon-treated fig fruit. Treatment was applied before the onset of ripening. Each value represents the mean of three biological replicates ± SE. I, inflorescence; R, receptacle.

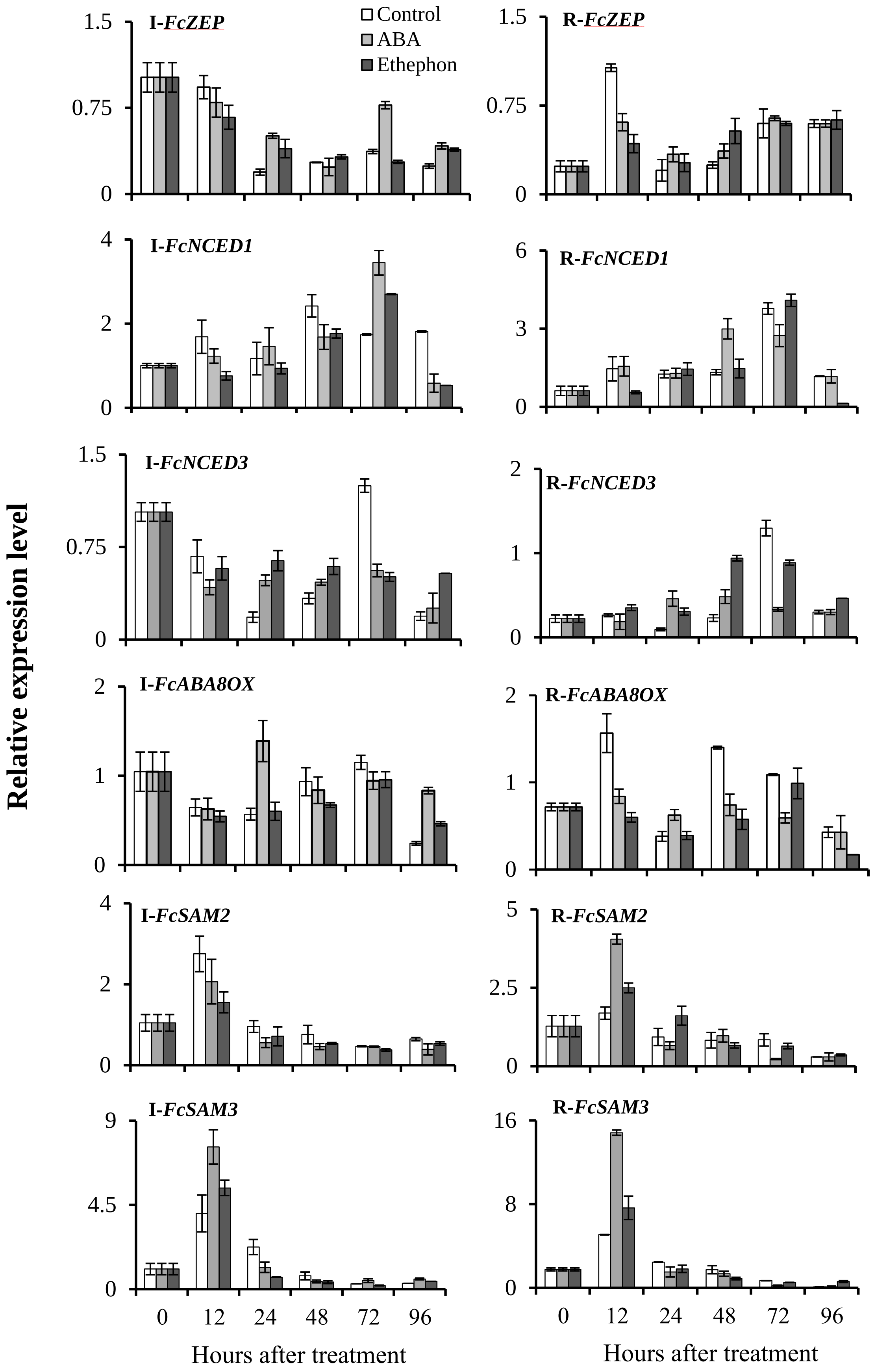

Fig. S2. Expression pattern of ABA-metabolism genes (*FcZEP, FcNCED1, FcNCED3* and *FcABA8OX*) and ethylene biosynthesis genes (*FcSAM2* and *FcSAM3*) in (A) fluridone-treated and (B) NDGA-treated fig fruit. Treatment was applied before the onset of ripening. Each value represents the mean of three biological replicates ± SE. I, inflorescence; R, receptacle.

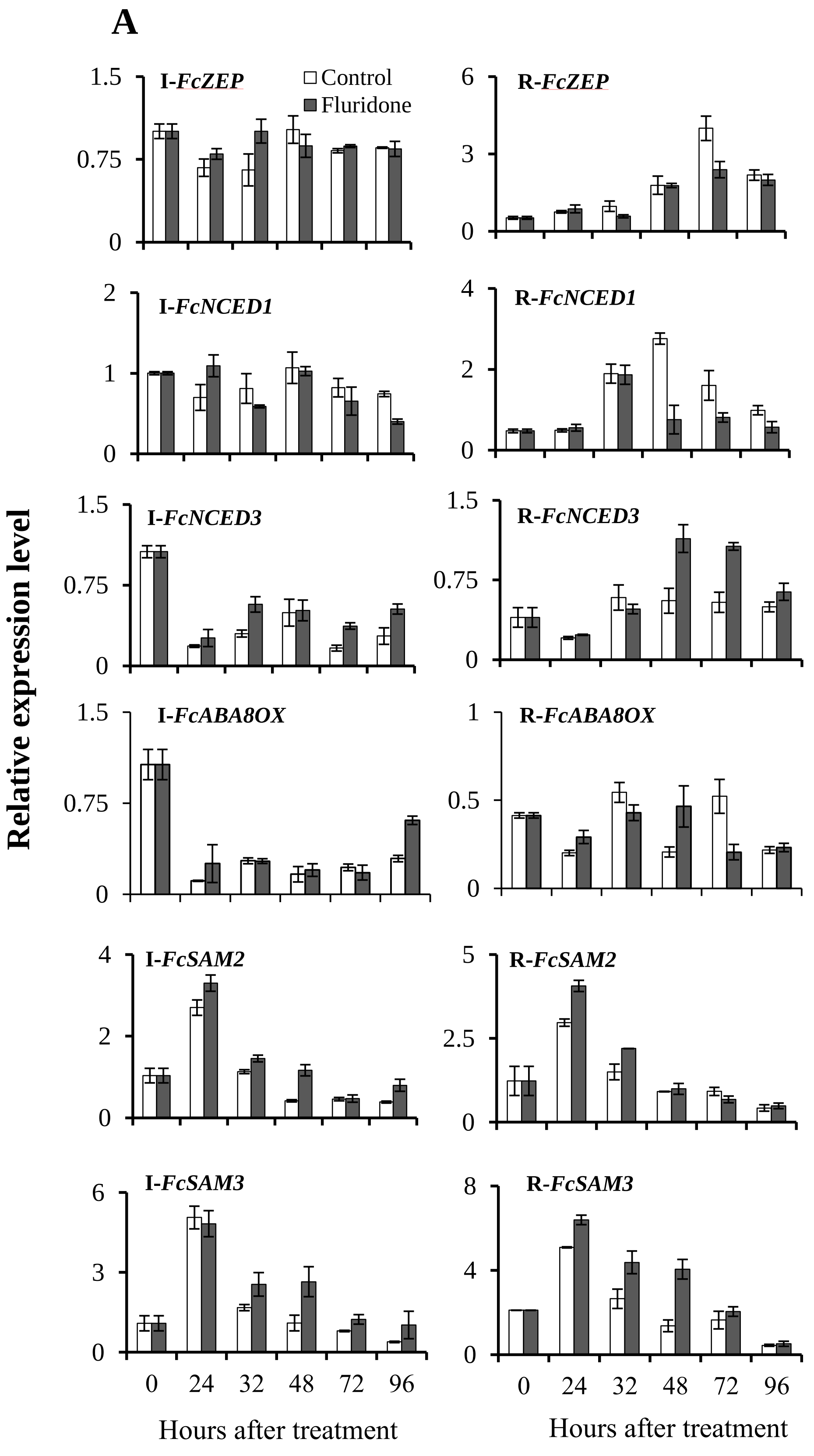

B

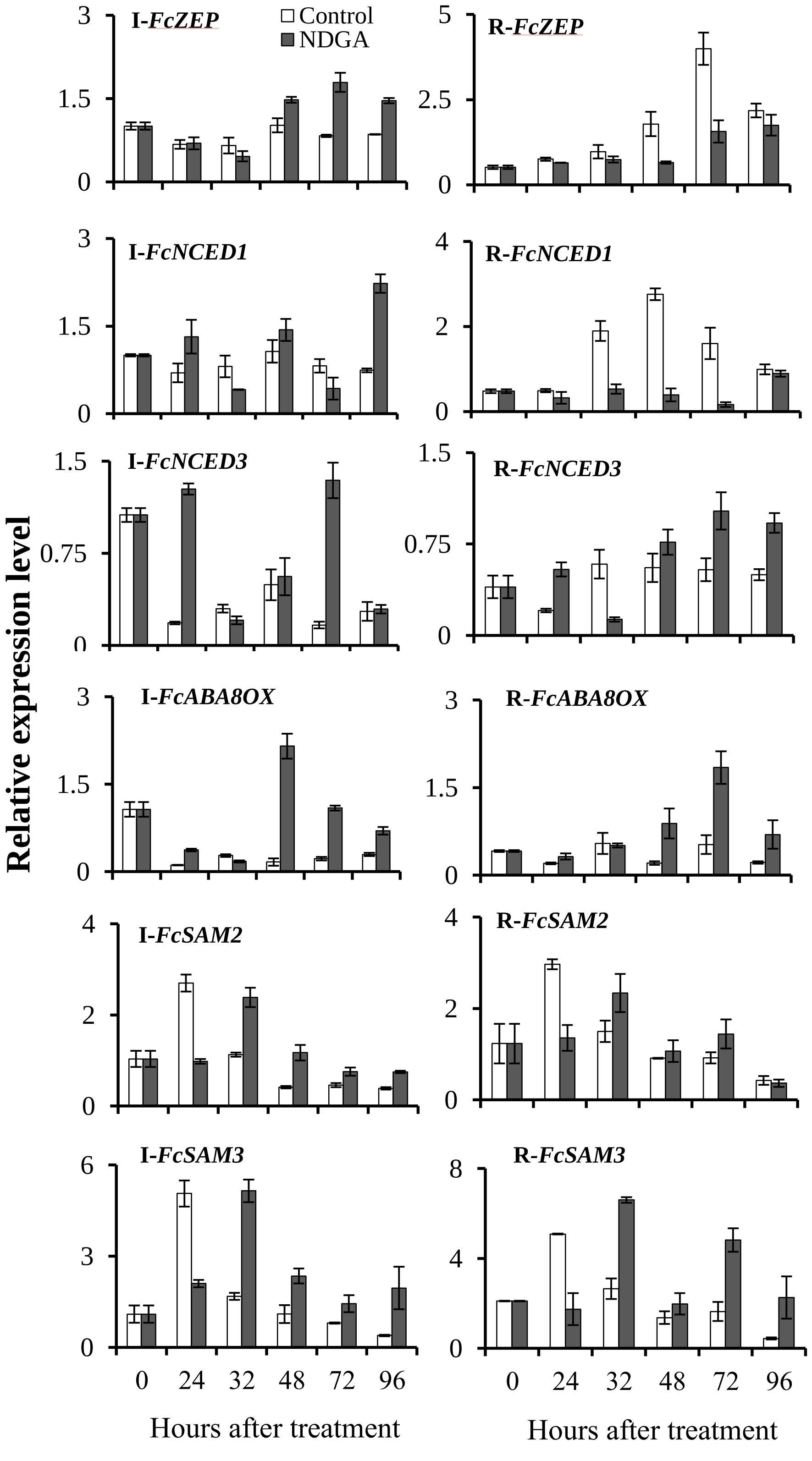

I-FcZEP
Control
NDGA
R-FcZEP
I-FcNCED1
R-FcNCED1
I-FcNCED3
R-FcNCED3
I-FcABA8OX
R-FcABA8OX
I-FcSAM2
R-FcSAM2
I-FcSAM3
R-FcSAM3
Relative expression level
0 24 32 48 72 96
Hours after treatment

Fig. S3. Expression pattern of MADS-box genes (*MADS1–7, 9–13*) following ABA and ethephon treatment in inflorescence and receptacle tissues.

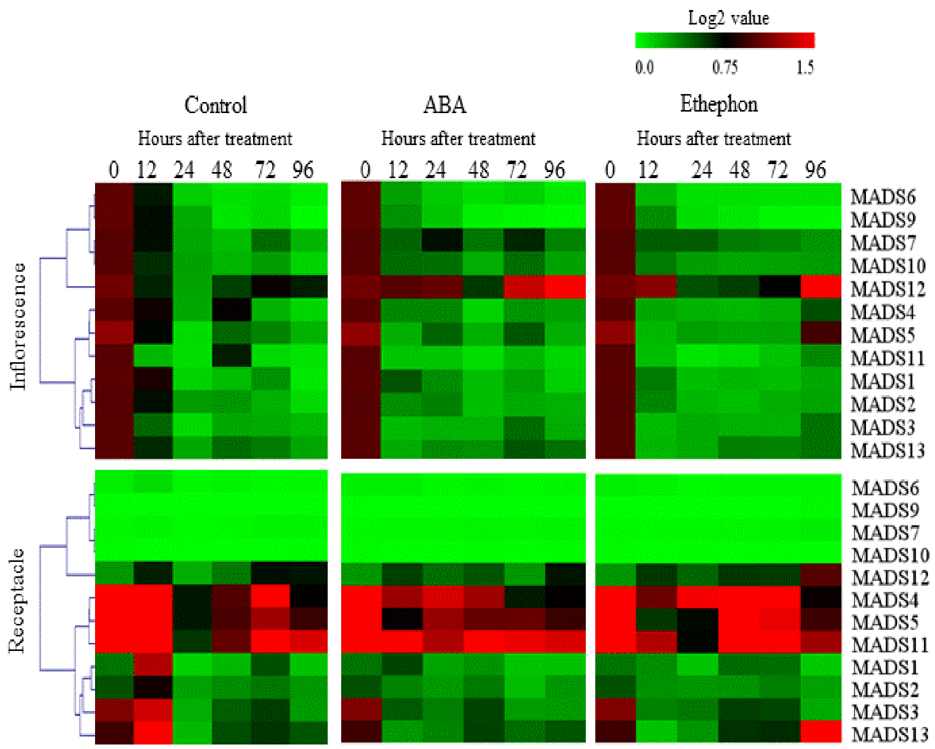

Fig. S4. Expression pattern of MADS-box genes (*MADS1–7, 9–13*) following (A) fluridone and (B) NDGA application in inflorescence and receptacle tissues.

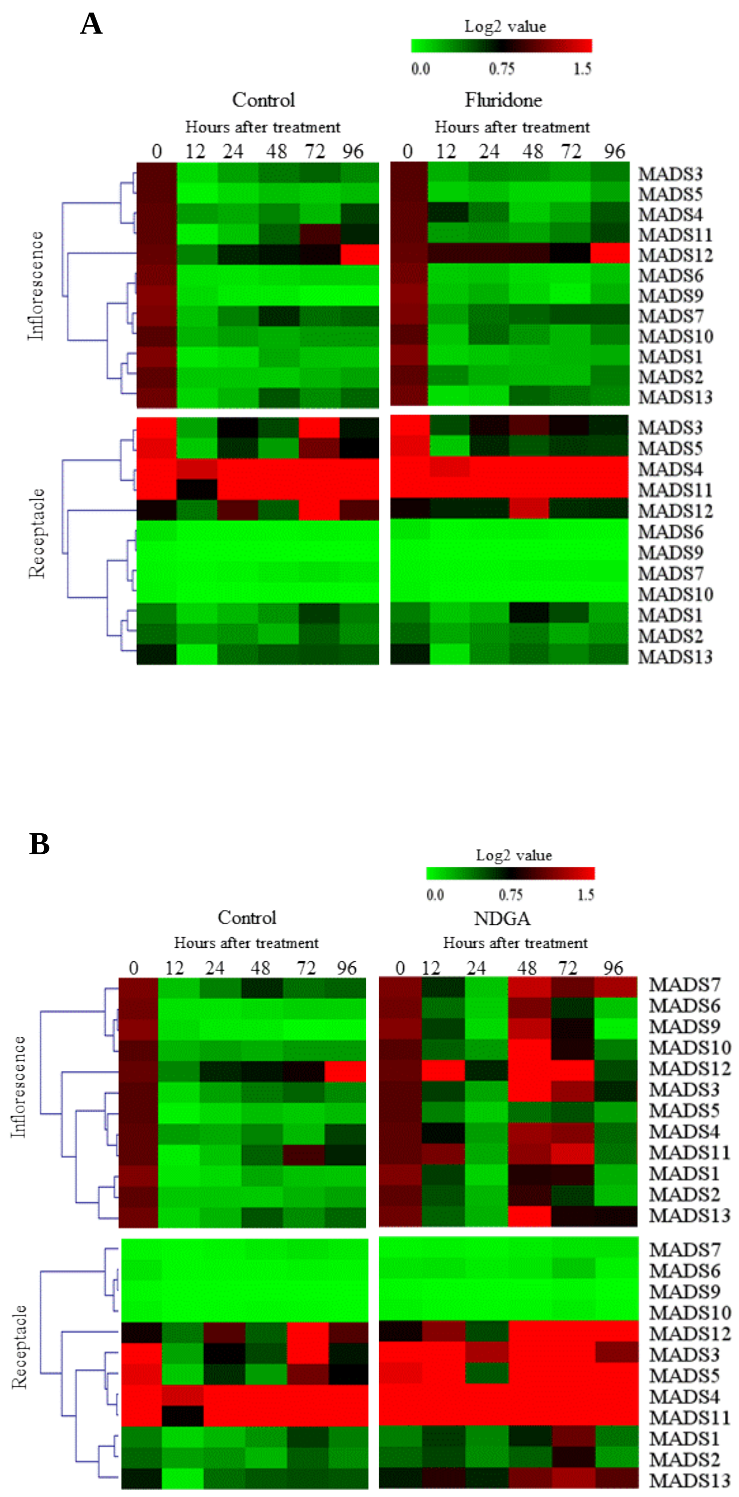

Fig. S5. Expression pattern of NAC genes (*NAC3, 4, 6–10)* following ABA and ethephon application in inflorescence and receptacle tissues.

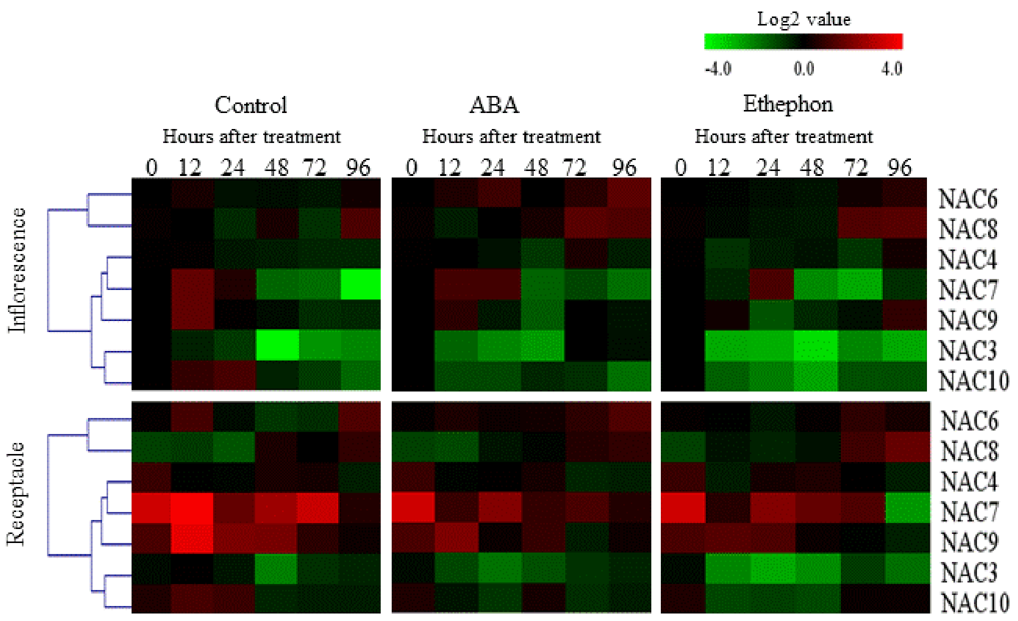

Fig. S6. Expression pattern of NAC genes (*NAC3, 4, 6–10*) following (A) fluridone and (B) NDGA application in inflorescence and receptacle tissues.

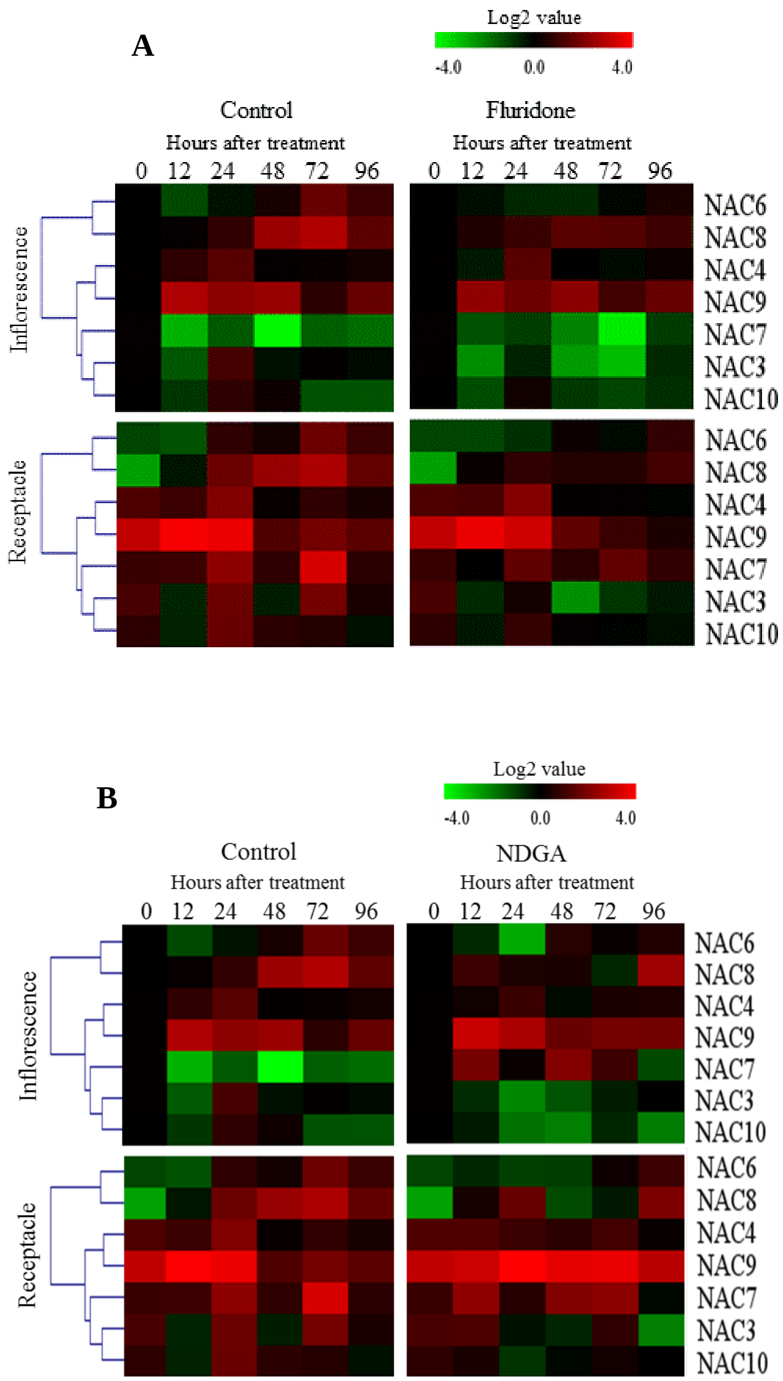

Fig. S7. Expression pattern of ERF genes following ABA and ethephon application in inflorescence and receptacle tissues.

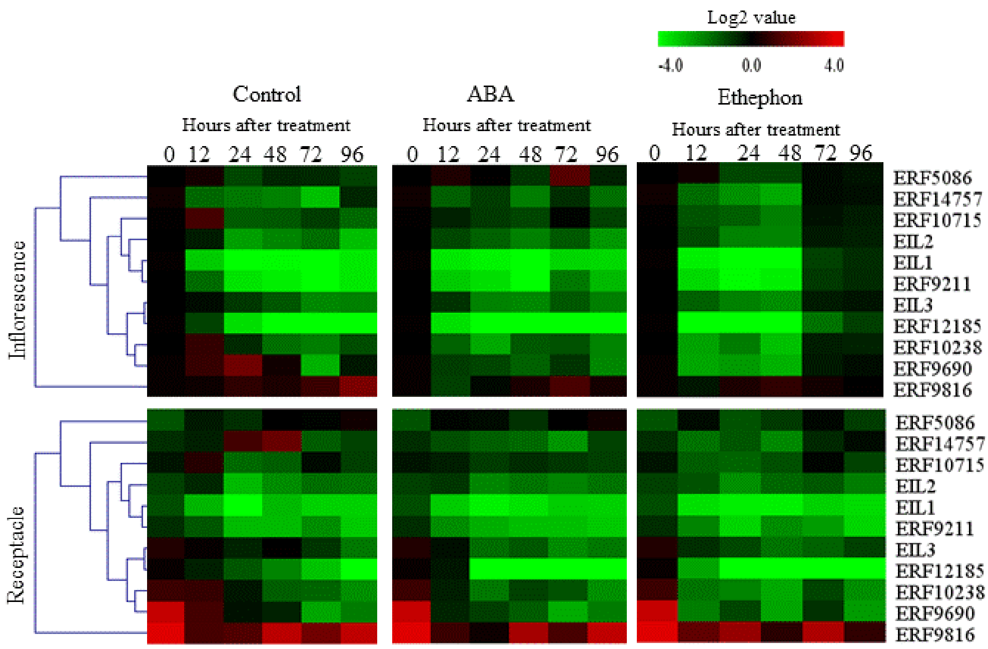

Fig. S8. Expression pattern of ERF genes following (A) fluridone and (B) NDGA application in inflorescence and receptacle tissues.

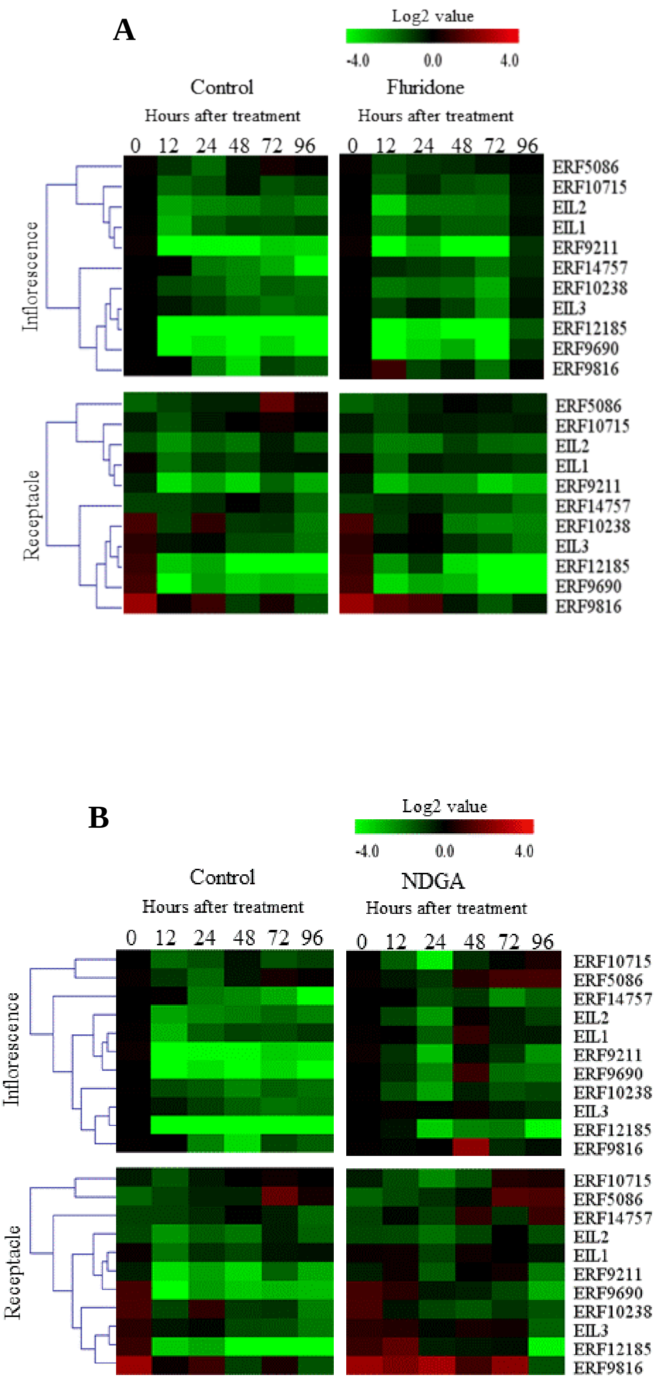

Fig. S9. Alignment of *FcNCED2* (Rosianski *et al.*, 2016a) amino acid sequence and *SlNCED1* protein (NCBI accession number NP_001234455.1). Black highlighted amino acid – identical residues; gray highlighted – similar residues. Alignment was performed by MUSCLE program using default parameters (http://www.ebi.ac.uk/Tools/msa/muscle) (Edgar, 2004).

```
FcNCED2      1 MASPPLAPNSSSWVELKKTTHTLSSSSSYLVDMGLGFSS-TISVKKKPN 49
SlNCED1      1 MATTT-SHATNTWIKTKLS---MPSS-------KEFGFASNSISLLKNQH 39

FcNCED2     50 ----TYTHCALHSPSVLHFPKQPYNQPV----ITKEPKKSHH--QHQQPP 89
SlNCED1     40 NRQSLNINSSLQAPPILHFPKQSSNYQTPKNNTISHPKQENNNSSSSTS 89

FcNCED2     90 QWSFLQRAAAMALDVAEGALISRERQTPLPKTADPRIQISGNFAPVPEQP 139
SlNCED1     90 KWNLVQKAAAMALDAVESALTKHELEHPLPKTADPRVQISGNFAPVPENP 139

FcNCED2    140 VHQDLPVTGTIPDCINGVYLRNGANPLFEPVAGHHFFDGDGMVHAVKIES 189
SlNCED1    140 VCQSLPVTGKIPKCVQGVYVRNGANPLFEPTAGHHFFDGDGMVHAVQFKN 189

FcNCED2    190 GSASYACRFTETQRLVQERELGRPVFPKAIGELHGHSGIARLLLFYARGL 239
SlNCED1    190 GSASYACRFTETERLVQEKALGRPVFPKAIGELHGHSGIARLMLFYARGL 239

FcNCED2    240 FGLLDHSHGTGVANAGLVYFNGRLLAMSEDDLPYHVRITNSGDLETVGRY 289
SlNCED1    240 FGLVDHSKGTGVANAGLVYFNNRLLAMSEDDLPYHVKVTPTGDLKTEGRF 289

FcNCED2    290 DFVEQLKSTMIAHPKVDPVSGEMFALSYDVVSKPYLKYFKFSANGEKSPD 339
SlNCED1    290 DFDGQLKSTMIAHPKLDPVSGELFALSYDVIQKPYLKYFRFSKNGEKSND 339

FcNCED2    340 VEIPLKVPTMMHDFAITQNFVVIPDQQVVFKLEEMVRGGSPVIYDKKKKS 389
SlNCED1    340 VEIPVEDPTMMHDFAITENFVVIPDQQVVFKMSEMIRGGSPVVYDKNKVS 389

FcNCED2    390 RFGILPKNSRDASDILWVESPETFCFHLWNAWEEPETEEVVVIGSCMTPA 439
SlNCED1    390 RFGILDKYAKDGSDLKWVEVPDCFCFHLWNAWEEAETDEIVVIGSCMTPP 439

FcNCED2    440 DSIFNECDESLKSVLSEIRLNLRTGQSTRRPIISEESEQVNLEAGMVNRN 489
SlNCED1    440 DSIFNECDEGLKSVLSEIRLNLKTGKSTRKSIIENPDEQVNLEAGMVNRN 489

FcNCED2    490 RLGRKTRFAYLAIAEPWPKVSGFAKVNLLTGEVKKYFYGDRRYGGEPFFL 539
SlNCED1    490 KLGRKTEYAYLAIAEPWPKVSGFAKVNLFTGEVEKFIYGDNKYGGEPLFL 539

FcNCED2    540 PKDQNDSETSEDDGYIMAFVHDERTGKSEMQIVNAVNLEMEAAVKLPSRV 589
SlNCED1    540 PRDPNSKE--EDDGYILAFVHDEKEWKSELQIVNAMSLKLEATVKLPSRV 587

FcNCED2    590 PYGFHGTFVDSKDLAFQAQE 609
SlNCED1    588 PYGFHGTFINANDLANQA-- 605
```

Fig. S10. Expression of ethylene-biosynthesis genes in the petiole of fig fruits at different developmental stages during ripening. Green – petioles from green/unripe fruit just before the start of ethylene production; 10–20%, 60% and 100% – petioles from fruit covered by the respective percentages of purple color. Each sample is composed of petioles of nine fruit and values represent means of three biological replications ± SE.

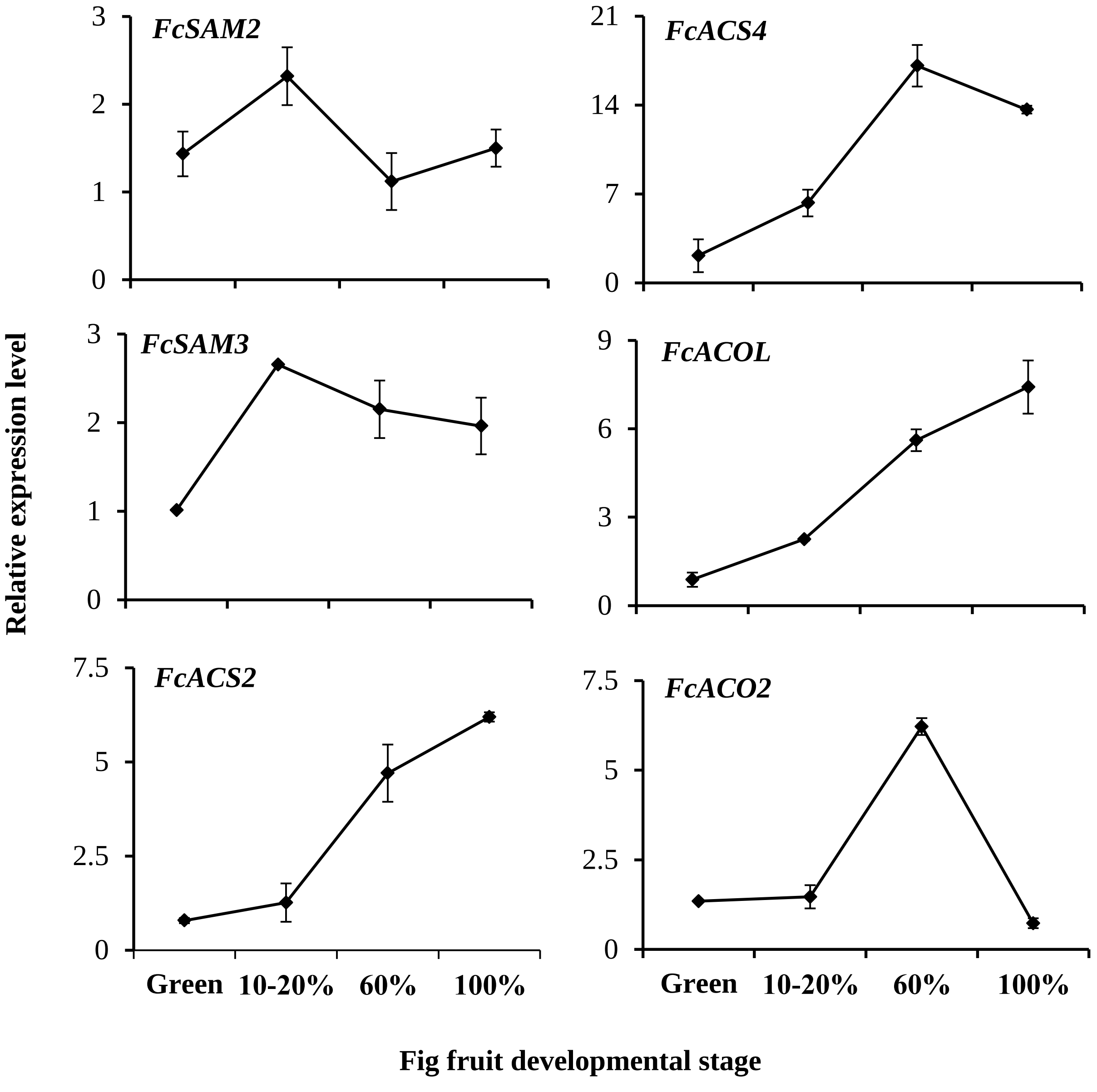

References

Edgar RC. 2004. MUSCLE: multiple sequence alignment with high accuracy and high throughput. *Nucleic Acids Research* **32**, 1792–1797.

Rosianski Y, Doron-Faigenboim A, Freiman ZE, Lama K, Milo-Cochavi S, Dahan Y, Kerem Z, Flaishman MA. 2016a. Tissue-specific transcriptome and hormonal regulation of pollinated and parthenocarpic fig (*Ficus carica* L.) fruit suggest that fruit ripening is coordinated by the reproductive part of the syconium. *Frontiers in Plant Science* **7,** 1696.
